# Phosphorylation of eIF4E Confers Resistance to Cellular Stress and DNA-Damaging Agents through an Interaction with 4E-T: A Rationale for Novel Therapeutic Approaches

**DOI:** 10.1371/journal.pone.0123352

**Published:** 2015-04-29

**Authors:** Alba Martínez, Marta Sesé, Javier Hernandez Losa, Nathaniel Robichaud, Nahum Sonenberg, Trond Aasen, Santiago Ramón y Cajal

**Affiliations:** 1 Molecular Pathology, Hospital Universitari Vall d’Hebron, Vall d'Hebron Institut de Recerca, VHIR, Universitat Autònoma de Barcelona, Barcelona, Spain; 2 Department of Pathology, Vall d’Hebron University Hospital, Barcelona, Spain; 3 McGill University, Department of Biochemistry, Goodman Cancer Research Centre, Montréal, Québec, Canada; Johns Hopkins University, UNITED STATES

## Abstract

Phosphorylation of the eukaryotic translation initiation factor eIF4E is associated with malignant progression and poor cancer prognosis. Accordingly, here we have analyzed the association between eIF4E phosphorylation and cellular resistance to oxidative stress, starvation, and DNA-damaging agents *in vitro*. Using immortalized and cancer cell lines, retroviral expression of a phosphomimetic (S209D) form of eIF4E, but not phospho-dead (S209A) eIF4E or GFP control, significantly increased cellular resistance to stress induced by DNA-damaging agents (cisplatin), starvation (glucose+glutamine withdrawal), and oxidative stress (arsenite). *De novo* accumulation of eIF4E-containing cytoplasmic bodies colocalizing with the eIF4E-binding protein 4E-T was observed after expression of phosphomimetic S209D, but not S209A or wild-type eIF4E. Increased resistance to cellular stress induced by eIF4E-S209D was lost upon knockdown of endogenous 4E-T or use of an eIF4E-W73A-S209D mutant unable to bind 4E-T. Cancer cells treated with the Mnk1/2 inhibitor CGP57380 to prevent eIF4E phosphorylation and mouse embryonic fibroblasts derived from Mnk1/2 knockout mice were also more sensitive to arsenite and cisplatin treatment. Polysome analysis revealed an 80S peak 2 hours after arsenite treatment in cells overexpressing phosphomimetic eIF4E, indicating translational stalling. Nonetheless, a selective increase was observed in the synthesis of some proteins (cyclin D1, HuR, and Mcl-1). We conclude that phosphorylation of eIF4E confers resistance to various cell stressors and that a direct interaction or regulation of 4E-T by eIF4E is required. Further delineation of this process may identify novel therapeutic avenues for cancer treatment, and these results support the use of modern Mnk1/2 inhibitors in conjunction with standard therapy.

## Introduction

Regulation of protein synthesis has recently been linked to a central role in cancer development and malignant progression. The eukaryotic translation initiation factor (eIF) 4E mediates association of the eIF4F complex (consisting of eIF4E, eIF4A, and eIF4G) with the 5'-methylated guanosine cap structure of mRNA and is an essential and rate-limiting factor of canonical protein synthesis initiation [1, 2]. eIF4E also contributes to nuclear-cytoplasmic export of certain mRNAs by binding a 50-nt element in the 3’UTR known as the eIF4E-sensitivity element (4E-SE) [3, 4]. Nuclear import of eIF4E is mediated by the transporter protein 4E-T (full name: eukaryotic translation initiation factor 4E nuclear import factor 1, EIF4ENIF1). 4E-T binds to eIF4E through a conserved binding motif (YXXXXLΦ) that is also found in eIF4G and in the family of translational suppressors known as eIF4E-binding proteins (4E-BPs) [5].

eIF4E is an oncogene with prognostic value in various human cancers, including head and neck squamous cell carcinoma and breast cancer [6–8]. *In vitro*, eIF4E induces malignant transformation through the regulation of multiple cell features, such as by increasing cell proliferation, cell survival, and anchorage-independence [6, 9, 10]. This is at least partly due to eIF4E enhancing the translation of mRNAs with long and highly structured 5' untranslated regions (UTRs), such as *C-MYC*, *BCL2*, *FGF2*, and *BIRC5* (survivin), referred to as eIF4E-sensitive mRNAs [11]. However, the exact molecular mechanisms of how eIF4E contributes to malignancy and, in particular, the role of eIF4E phosphorylation remain unclear.

Phosphorylation of eIF4E at Ser-209 by the kinases Mnk1 and Mnk2 in response to mitogens, tumor promoters, and growth factors [12–15] is critical for its oncogenic activity [16]. Phosphorylation of eIF4E appears to selectively control the translation of a subset of mRNAs that encode proliferation and pro-survival proteins (such as BIRC2 and Mcl-1), several paracrine factors involved in inflammation (Smad2, the chemokines CCL2, CCL7, and CCL9), extracellular matrix proteins (MMP3, MMP9), and proteins related to angiogenesis (VEGFC) [17]. Moreover, phosphorylated eIF4E seems to be involved in export of a set of RNAs from the nucleus to the cytoplasm (including cyclin D1, HDM2, and ODC) and has been related with a weak affinity for capped RNA. This lower affinity probably allows mRNA release and confers a faster turnover of certain RNAs [18, 19].

The oncogenic features mediated through eIF4E phosphorylation have also been analyzed in *in vivo* models. In the Eu-myc mouse lymphoma model [16, 20, 21], expression of wild-type eIF4E, the phosphomimetic eIF4E-S209D mutant, or activation of the eIF4E kinase Mnk1 all accelerated tumor development. In contrast, the phospho-null mutant S209A or dominant negative Mnk1/2 suppressed lymphomagenesis [16]. In PTEN-null mouse lymphoma and prostate cancer models, disruption of eIF4E phosphorylation abrogates tumor development. Similar results were observed in mice harboring knockout (KO) Mnk1/2 genes. Curiously, Mnk1/2 KO mice do not exhibit any conspicuous phenotype, indicating that phosphorylation of eIF4E is not required for normal tissue function or development [17]. It should be noted however, that the use of different cell lines or assay type appears to be important. Topisirovic *et al*. for example, who used mouse NIH3T3 cells in an anchorage-independent soft agar assay, only observed an effect upon overexpression of wild type eIF4E but not the phosphomimetic mutant eIF4E-S209D [22].

Various cytoplasmic bodies are formed when cells are stressed, such as stress granules (SGs) and processing bodies (also known as P-bodies; PBs). Although eIF4E and 4E-T have been described as components of both SGs and PBs, the role of eIF4E phosphorylation in the formation of these intracellular structures has not been sufficiently studied. Curiously, arsenite treatment induces both phosphorylation of eIF4E and assembly of PBs and SGs, and results in physically associated SG-PB structures [23]. SGs sequester mRNAs in a protein complex that includes preinitiation and translation-related factors and mRNA-binding proteins and which can act as a site for mRNA storage and subsequent re-entry to translation. On the other hand, mammalian PBs contain components of the 5’ to 3’ decay machinery, nonsense-mediated decay pathways, RNA-induced silencing machinery, and activators of mRNA decay pathways [24]. However, some protein components of PBs are also implicated in translational repression, and mRNAs that are associated with PBs may re-enter translation either directly or through SGs [25, 26].

The relationship between the phosphorylation of eIF4E and cellular stress, survival, and malignant progression remains to be studied in detail. Here, we demonstrate that phosphorylation of eIF4E at Ser-209 increases resistance to cellular stress, including oxidative stress (arsenite), starvation (glucose/glutamine), and cytotoxic stress (cisplatin), leading to enhanced cell survival and subsequent cell recovery, proliferation, and tumor progression. This process appears to act through an interaction with 4E-T and a qualitative regulation of protein synthesis, processes that could potentially be exploited therapeutically to target certain cancers.

## Results

### Phosphorylation of eIF4E augments clonogenic formation ability and resistance to oxidative stress, nutrient starvation, and DNA-damaging chemotherapeutic drugs

**Fig 1:**
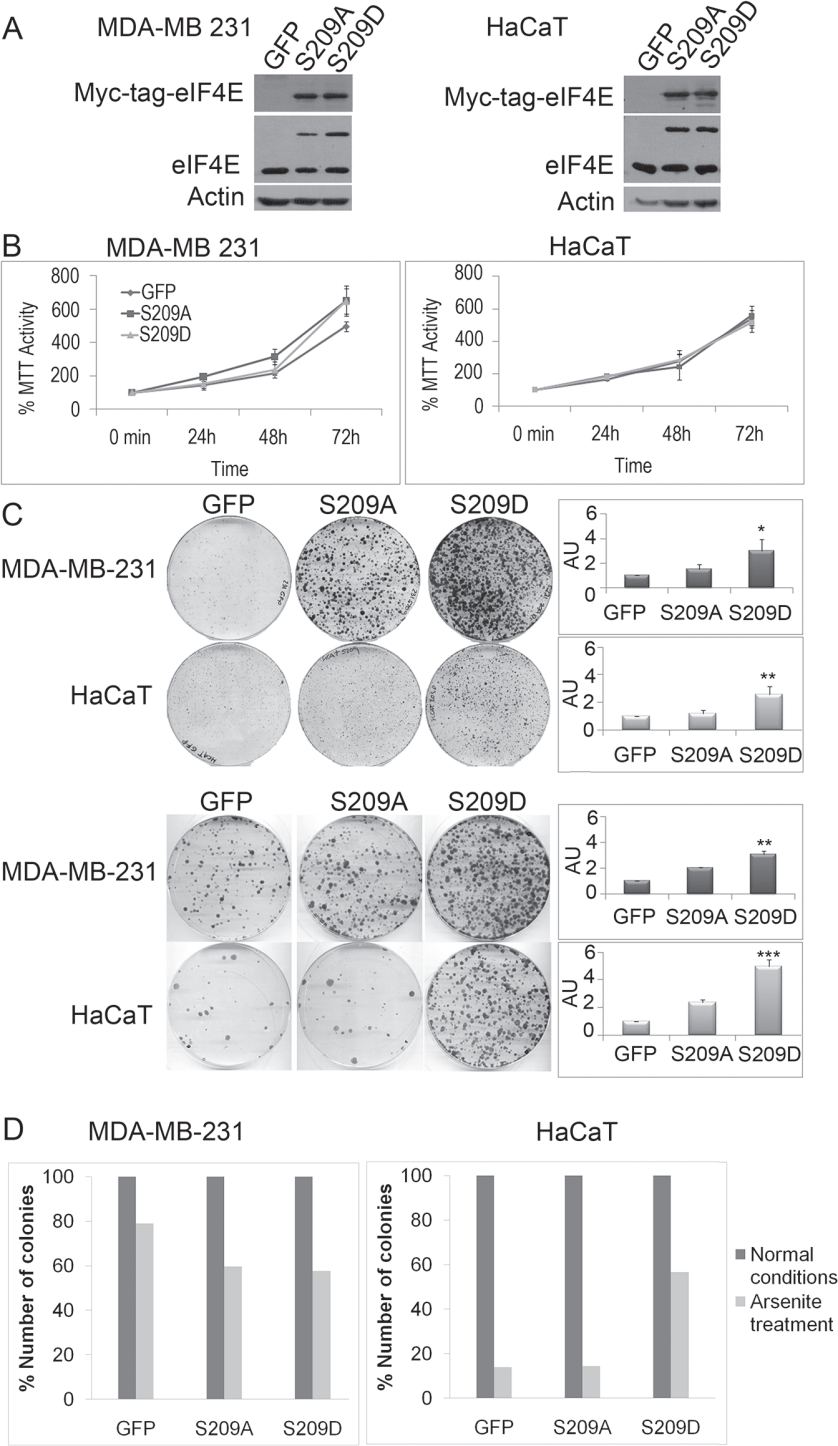
Overexpression of phosphomimetic eIF4E-S209D does not affect proliferation but increases clonogenic cell survival. A, Western blot analysis showed similar, moderate levels of Myc-tagged exogenous eIF4E-S209D or-S209A and endogenous eIF4E expression in MDA-MB-231 and HaCaT cell lines. B, MTT cell proliferation assays revealed no significant difference in cell growth between GFP—or eIF4E-mutant–expressing cells under normal conditions. C, Crystal violet staining of clonogenic colony formation assays clearly indicate an increase in both colony number and size upon eIF4E-S209D expression compared with GFP control cells, which is reflected by a statistically significant increase in the total number of stained cells as measured by overall crystal violet absorbance. A moderate but not statistically significant increase in eIF4E-S209A–expressing cells was noted. Graphs depict the overall cell growth as measured in a crystal violet absorption assay. The survival and growth advantage after expression of eIF4E-S209D is highly significant in both HaCaT and MDA-MB-231 cells treated with arsenite for 90 minutes to induce oxidative stress before being plated. D, Graphs representing the percentage of the number of colonies after arsenite treatment compared to normal conditions in each case. Arsenite treatment clearly decreases the number of colonies in both cell lines. * = P<0.05, ** = P<0.01 and *** = P<0.001, compared to control, n = 3. (AU: Absorbance Units)

Phosphorylation of eIF4E Ser-209 by Mnk1/2 is known to affect cell proliferation and tumor malignancy [27]. To further understand the role of eIF4E phosphorylation in these processes, we infected immortalized and malignant cell lines with retrovirus to overexpress GFP (control) or Myc-tagged phosphomimetic (S209D) or phospho-dead (S209A) versions of eIF4E. The levels of protein expression between the two mutants were similar, and were approximately the same as that of endogenous eIF4E in MDA-MB-231 and HaCaT cells, indicating a physiologically relevant expression system (Fig 1A). Analysis of cell proliferation did not reveal any statistically significant differences between the three expression constructs (Fig 1B).

In an anchorage-dependent clonogenic assay, however, expression of S209D (and to a lesser extent S209A) clearly increased both colony size and number in MDA-MB-231 malignant breast cancer cells as well as in non-tumorigenic HaCaT keratinocytes (Fig 1C and S1A Fig), consistent with previous reports [16]. In our control experiments, overexpression of eIF4E WT also increased the number and size of the colonies in both cell lines, comparable to the S209D mutant (S1 Fig). Similar results were observed in other cell lines, including MDA-MB-468 breast cancer cells (S1D Fig).

Since clonogenic assays subject cells to stress conditions, we reasoned that phosphorylation of eIF4E confers a positive survival advantage during and/or after stress situations. We therefore treated the cells before plating for 90 minutes with arsenite, a well-known inducer of oxidative stress [28]. As expected, the overall number of colonies was reduced (S1A Fig), but we still observed a statistically significant increase in colony formation in cells expressing phosphomimetic S209D (Fig 1D and S1A Fig). Curiously, although we observed fewer colonies after arsenite treatment, the surviving colonies were bigger compared to untreated conditions (Fig 1D), which may be due to endogenous phosphorylation of eIF4E, enhanced migration and cell clustering, or perhaps reduced nutrient competition due to fewer surviving colonies following arsenite treatment.

**Fig 2:**
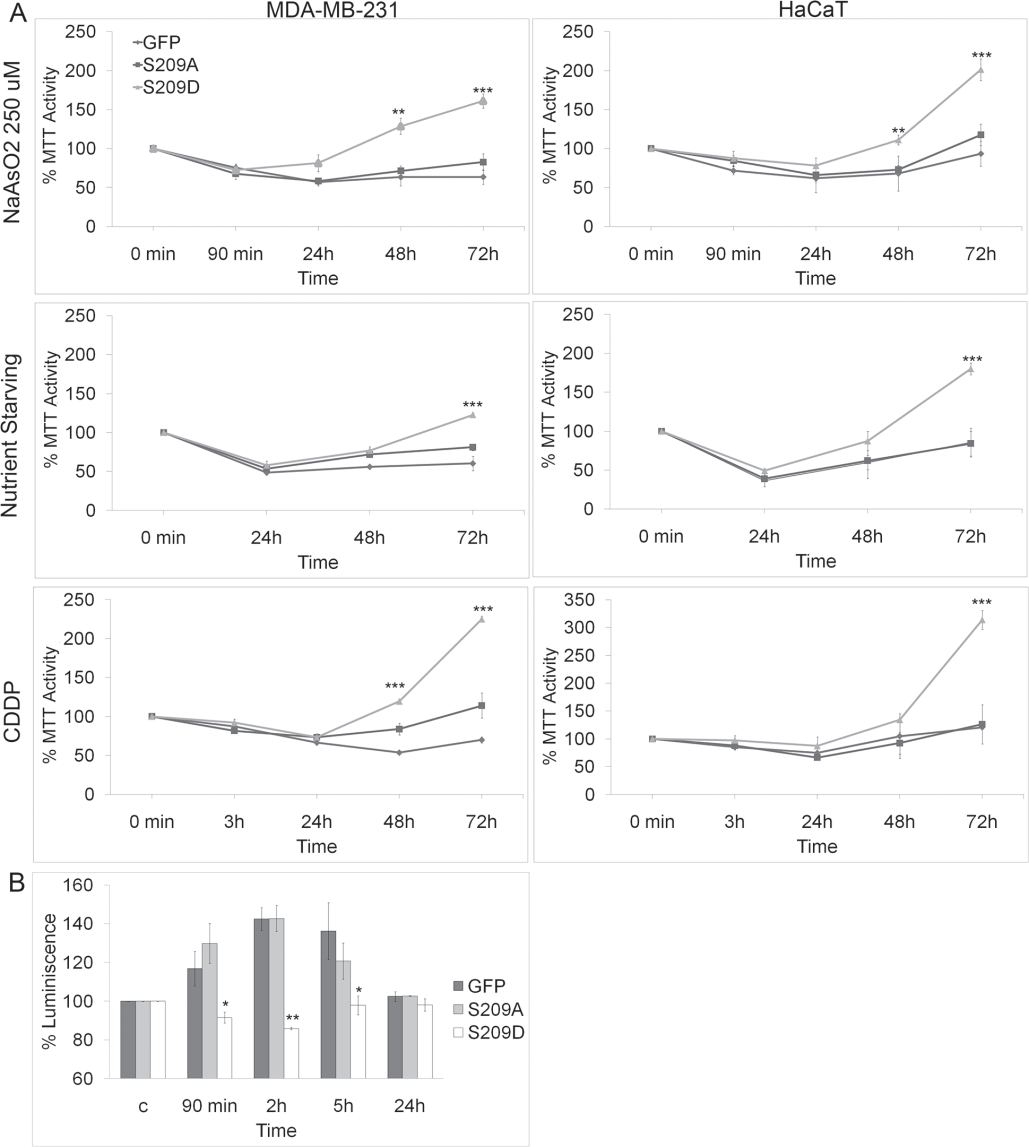
MDA-MB-231 and HaCaT cells show increased resistance to stress after overexpression of phosphomimetic eIF4E. A, MDA-MB-231 and HaCaT cells were subjected to either arsenite (NaAsO_2_), nutrient starvation, or cisplatin (CDDP) treatment, and cell viability was measured by an MTT assay after 24, 48, and 72 hours. In all cases, eIF4E-S209D significantly increased cell viability. B, Apoptotic activity measured by a caspase-3/-7 luminescence assay. Significant activation of caspase-3/-7 activity was observed following arsenite treatment in eIF4E-S209A– and GFP–expressing cells, which was completely prevented by eIF4E-S209D. * = P<0.05, ** P<0.01 and *** = P<0.001 compared to control, n = 3.

For a more detailed analysis, we assessed proliferation and viability after exposing cells to a variety of stress signals: arsenite (250 μM NaAsO_2_ for 90 minutes); cisplatin (33 μM CDDP for 3 hours), a DNA-damaging chemotherapeutic agent; and nutrient starvation (lack of glucose and glutamate for 24 hours). Cell viability and recovery after restoration to normal culture conditions was measured at 24, 48, and 72 hours. As seen in Fig 2A, both MDA-MB-231 and HaCaT cells overexpressing S209D clearly showed increased resistance and recovery to oxidative, nutrient, and DNA damage stress conditions compared with control cells. Similar results were observed in various other cancer cell lines, including MDA-MB-468 and HeLa cells (S2 Fig). To further validate the importance of eIF4E in resistance to stress, and to confirm that the recovery is fully due to the overexpression of a functional S209D mutant, without the involvement of endogenous phosphorylated eIF4E, we transfected the MDA-MB-231 cells with a short hairpin construct known to target the 3’UTR of endogenous eIF4E without affecting exogenous eIF4E (S3A Fig) [29, 30]. Reducing endogenous eIF4E, without affecting regular cell growth, significantly reduced the capacity of cells to recover from arsenite (S3B Fig). In this setting, exogenous eIF4E-S209D was still completely sufficient to rescue cells from this stress. Curiously, S209A also moderately aided recovery, probably by rescuing some of the general functions of endogenous unphosphorylated eIF4E. The effects were less obvious with cisplatin treatment, although again, S209D efficiently improved cellular resistance comparable to cells without endogenous eIF4E knockdown (S3C Fig).

Cellular stress, including oxidative stress from arsenite, is known to induce apoptosis [31]. In addition, it has been demonstrated in mouse models that phosphorylated eIF4E mainly promotes tumorigenesis by suppressing apoptosis [20]. In accordance with the previous results, expression of S209D—but not S209A or GFP—completely prevented arsenite-induced apoptosis in MDA-MB-231 cells, as measured by caspase-3/-7 activation (Fig 2B). These results suggest that the presence of phosphorylated eIF4E at a time of stress provides an additional survival advantage.

### Prevention of endogenous eIF4E phosphorylation increases the sensitivity of cells to stress *in vitro*

**Fig 3:**
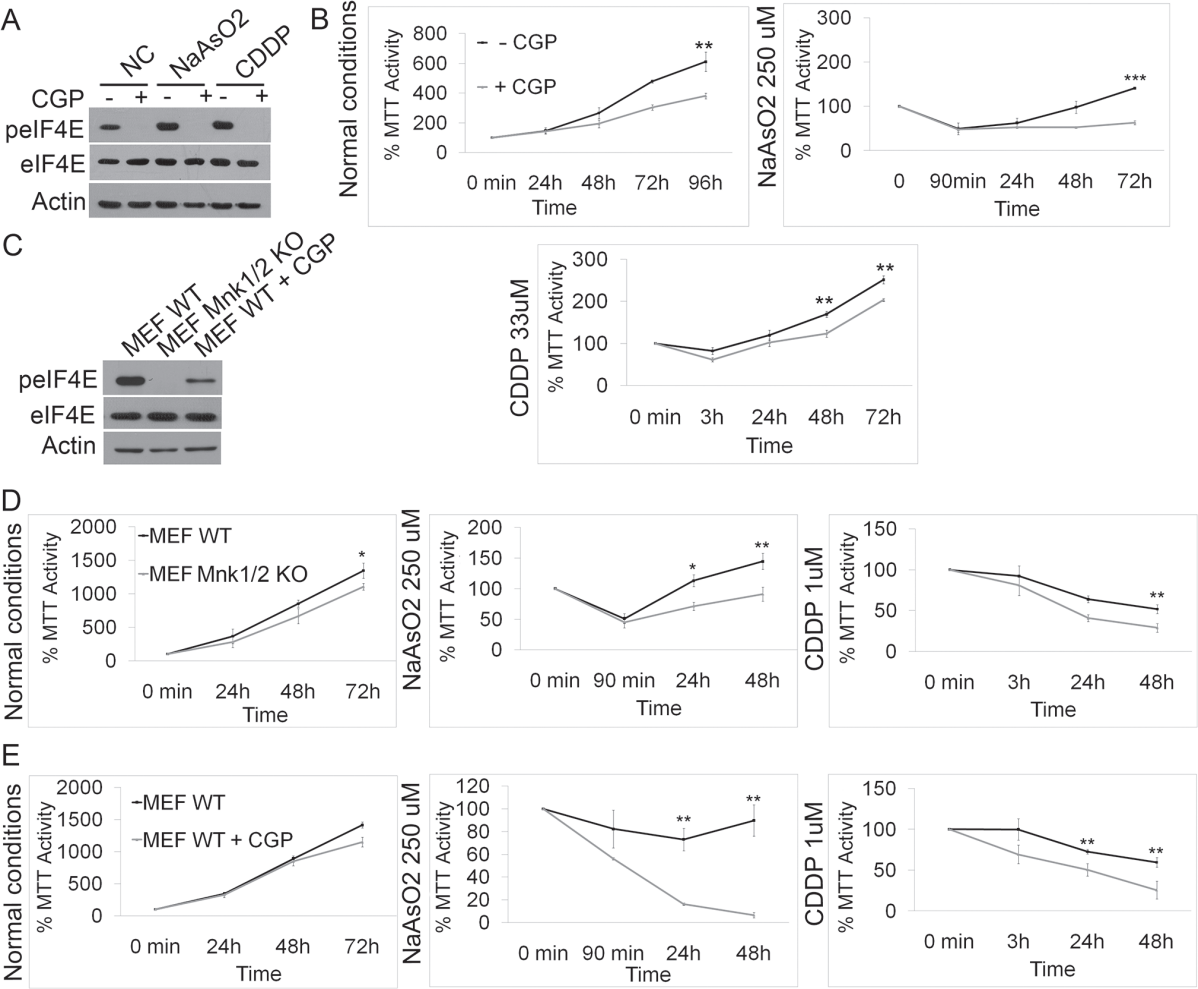
Phosphorylation of endogenous eIF4E increases cellular resistance to stress. A, Expression of phospho-eIF4E with and without CGP57380 treatment and with arsenite or cisplatin treatment in MDA-MB-231 cells. B, MDA-MB-231 cells treated with CGP57380 to inhibit Mnk1/2-mediated phosphorylation of eIF4E displayed slightly decreased cell proliferation under normal conditions, whereas cell recovery after oxidative stress by arsenite (and to less extent cisplatin) was dramatically reduced. C, Western blotting showed a lack of phospho-eIF4E in MEFs from Mnk1/2 null mice and significantly reduced phospho-eIF4E in wild-type MEFs treated with CGP57380. D, MTT growth assays demonstrated a slight decrease in the proliferation of MEFs from Mnk1/2 null mice compared to wild-type MEFs and a significant reduction in cell recovery following either arsenite or cisplatin treatment. E, Inhibiting Mnk1/2 in wild-type MEFs with CGP 57380 significantly reduced cellular recovery from arsenite or cisplatin treatment. * = P<0.05, ** = P<0.01 and *** P = <0.001 compared to control, n = 3.

To corroborate these data, we treated cells with the Mnk inhibitor CGP 57380, which effectively blocks phosphorylation of endogenous eIF4E (Fig 3A) [32]. MDA-MB-231 cells express phosphorylated eIF4E under normal conditions (Fig 3A) and blocking Mnk1/2-mediated eIF4E phosphorylation with CGP 57380 indeed reduce proliferation slightly, reaching significance at 96h. Blocking Mnk1/2 significantly reduces cell viability already after 48 hours upon treatment with cell stressors such as arsenite and cisplatin (Fig 3B). We performed a similar analysis in mouse embryonic fibroblasts (MEFs) isolated from wild-type, as well as from Mnk1/2 KO mice that are unable to phosphorylate eIF4E (Fig 3C). Although having a very slight reduced growth rate under normal conditions, Mnk1/2 KO MEFs were significantly less resistant to both arsenite and cisplatin treatment compared to wild-type MEFs (Fig 3D). Concordantly, a significant reduction in viability was observed in wild-type MEFs when blocking eIF4E phosphorylation with CGP 57380 treatment (Fig 3E). To control that the major effect of CGP 57380 treatment was specific and via Mnk1/2, we treated Mnk1/2 KO MEFs with or without CGP 57380, expecting little additional effect. Indeed, no significant additional loss of resistance was observed in the presence of the inhibitor in these cells (S4A Fig), strongly suggesting the inhibitor is acting mainly via Mnk1/2. As an additional control experiment, we reduced Mnk1 expression in MDA-MB-231 cells with a specific shRNA, which lead to loss of eIF4E phosphorylation (S4B Fig). Similar to CGP 57380 treatment, this lead to a reduced resistance to arsenite, and indeed supplementation with the inhibitor under these conditions did not significantly enhance this effect (S4C Fig). This suggests the effect of CGP 57380 is fairly specific and that the Mnk1/2 pathway is important for stress resistance.

Thus, from these observations, in conjunction with our phosphomimetic overexpression data, we conclude that phosphorylated eIF4E by Mnk1/2 likely enhances cellular resistance to stress and subsequently increases cell recovery after stress removal.

### Phosphomimetic eIF4E forms *de novo* cytoplasmic bodies and increased affinity for 4E-T under normal conditions

**Fig 4:**
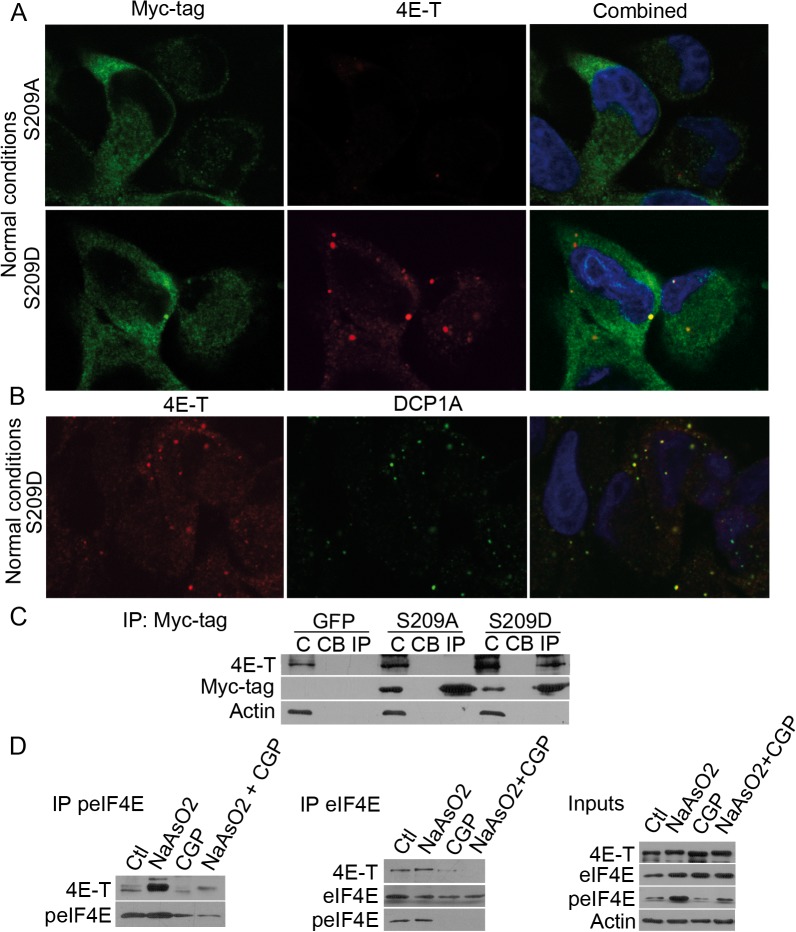
Phospho-eIF4E interacts with 4E-T and induces *de novo* cytoplasmic bodies. A, Confocal immunofluorescence analysis of MDA-MB-231 cells demonstrated *de novo* formation of cytoplasmic 4E-T bodies after overexpression of eIF4E-S209D, with partial colocalization. Formation of cytoplasmic 4E-T bodies was not observed upon overexpression of eIF4E-S209A. B, The 4E-T bodies in the eIF4E-S209D–expressing cells colocalized with a marker of P-bodies (DCP1A) in normal conditions. C, Immunoprecipitation assays with anti-Myc-tag antibodies against eIF4E-S209A or-S209D demonstrated a specific interaction between phosphomimetic eIF4E-S209D and 4E-T. D, Arsenite treatment increased the phosphorylation of endogenous eIF4E and dramatically increased the interaction between eIF4E and 4E-T, whereas treatment with CGP57380 reduced phospho-eIF4E and significantly diminished the amount of 4E-T interacting with eIF4E. 10% inputs used in the immunoprecipitations is shown on the side.

eIF4E, as well as the eIF4E transporter 4E-T, has been shown to colocalize with markers of PBs, important regulators of mRNA stability and translation during stress [33]. Surprisingly, we observed that the expression of phosphomimetic eIF4E-S209D, but not the control wild-type or S209A mutant, caused *de novo* formation of cytoplasmic bodies in MDA-MB-231 cells—even in the absence of external stress. These *de novo* formed bodies partially colocalized with the eIF4E-binding protein 4E-T (Fig 4A). We show that in most of the cells where these cytoplasmatic bodies appear, S209D colocalize with 4E-T (S5A Fig). These *de novo* forming bodies colocalizing to 4E-T were consistently observed in other cell line tested including HaCaT cells (S5B Fig). In control experiments we also show (as expected) that phosphorylation of endogenous eIF4E in MDA-MB-231 by arsenite treatment induces colocalization of peIF4E and 4E-T in cytoplasmic bodies, further indicating that this observation may be driven by the phosphorylation of eIF4E (S5C Fig). To determine the localization of 4E-T better, and to discard any non-specific staining or background, we used three different antibodies. Either using the S209D mutant or endogenous peIF4E we observed the cytoplasmic bodies in all cases. To determine whether these *de novo* formed bodies are PBs or SGs, we examined the interaction between 4E-T and markers of PBs (DCP1A) and SGs (TIA-1). In cells overexpressing the S209D mutant, endogenous 4E-T formed novel cytoplasmic bodies that colocalized with the PB marker DCP1A, but not the SG marker TIA-1, confirming previous results that 4E-T can localize to PBs (Fig 4B and data not shown).

To confirm the direct eIF4E/4E-T interaction, we immunoprecipitated exogenous eIF4E with an anti-Myc antibody in MDA-MB-231 cells. Strikingly, eIF4E-S209D, but not-S209A, was able to pull down 4E-T under normal conditions, suggesting that phosphorylation of eIF4E increases its affinity for or proximity to 4E-T (Fig 4C). To corroborate these data with endogenous eIF4E, we treated the MDA-MB-231 cell line with arsenite (inducing eIF4E phosphorylation) with or without CGP 57380 treatment (to prevent eIF4E phosphorylation). Arsenite-induced phosphorylation increased the interaction between eIF4E and 4E-T (as shown by increased pull-down with the phospho-specific antibody of eIF4E and total eIF4E), whereas CGP 57380 reduced this interaction (Fig 4D), confirming our results obtained with the phosphomimetic overexpression studies. Total inputs also indicate that arsenite induces phosphorylation of eIF4E, which is prevented by CGP 57380, as expected, with no change in total eIF4E (Fig 4D). Finally, we tested the affinity between eIF4E and 4E-T comparing normal MEFs and Mnk1/2 KO MEFs that lack phosphorylated eIF4E (S6 Fig). This indicated (at least under normal condition where we observe formation of the *de novo* bodies) an increased interaction between eIF4E and 4E-T in wild type cells with phosphorylated eIF4E. From these observations, we conclude that phosphorylation of eIF4E is implicated in some aspects controlling its binding with 4E-T and that this complex is required for the formation of *de novo* cytoplasmic bodies.

### Interplay between 4E-T and phospho-eIF4E confers resistance to cellular stress

**Fig 5:**
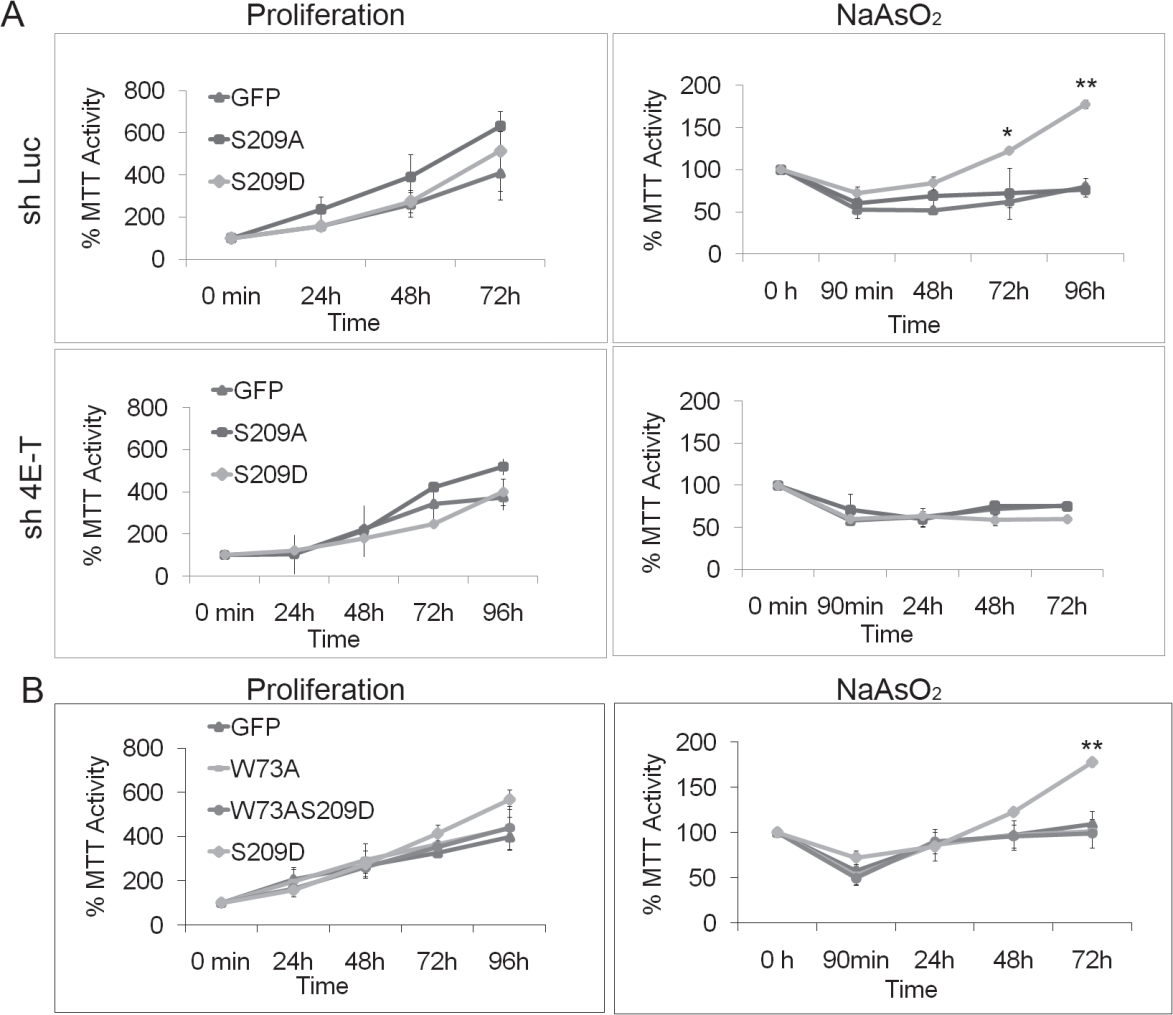
Phospho-eIF4E/4E-T binding is necessary for recovery after stress. A, Cells stably expressing eIF4E-S209D or-S209A were transfected with scramble or specific short hairpins to knockdown 4E-T expression. eIF4E-S209D significantly enhanced cell recovery from arsenite stress in scramble-transfected cells, whereas knockdown of 4E-T completely abolished any protective effect of eIF4E-S209D. B, Expression of the eIF4E mutants W73A, W73A/S209D, both unable to bind 4E-T, and S209D; W73A and W73A/S209D did not confer any resistance to arsenite treatment compared with S209D. * = P<0.05 and ** = P<0.01 compared to control, n = 3.

We speculated that the association between 4E-T and phosphorylated eIF4E might regulate resistance to stress. We therefore analyzed the role of the eIF4E/4E-T complex in MDA-MB-231 cells by knocking down 4E-T and by using eIF4E mutants unable to bind 4E-T. 4E-T protein levels were downregulated by over 80% following transfection with shRNA plasmids targeting 4E-T (S7 Fig). Knockdown of 4E-T in MDA-MB-231 cells did not affect proliferation (Fig 5A). However, it completely abolished the added resistance to arsenite mediated by eIF4E-S209D (Fig 5A). It thus appears that 4E-T is crucial for the protection against cellular stress conferred by phosphorylated eIF4E. To further delineate the hypothesis that a specific interplay between phospho-eIF4E and 4E-T is required for stress resistance, we used the eIF4E-S209D-W73A mutant that does not bind 4E-T ([4] and S8 Fig). The inability of eIF4E to interact with 4E-T completely abolished the increased resistance to stress mediated by the S209D mutant (Fig 5B). It should be noted that this mutant also do not bind eIF4G and initiates translation however. On the other hand, it does retain its ability to transport mRNA from the nucleus to the cytoplasm, suggesting mRNA export is not the mechanism of enhanced resistance. Overall, the results from our 4E-T knockdown experiments, supported by result where the eIF4E-W73A mutants unable to bind 4E-T, strongly support the theory that an association between 4E-T and the eIF4E protein is crucial for stress resistance.

### Phosphomimetic eIF4E mediates a rapid and dynamic regulation of protein synthesis after stress

**Fig 6:**
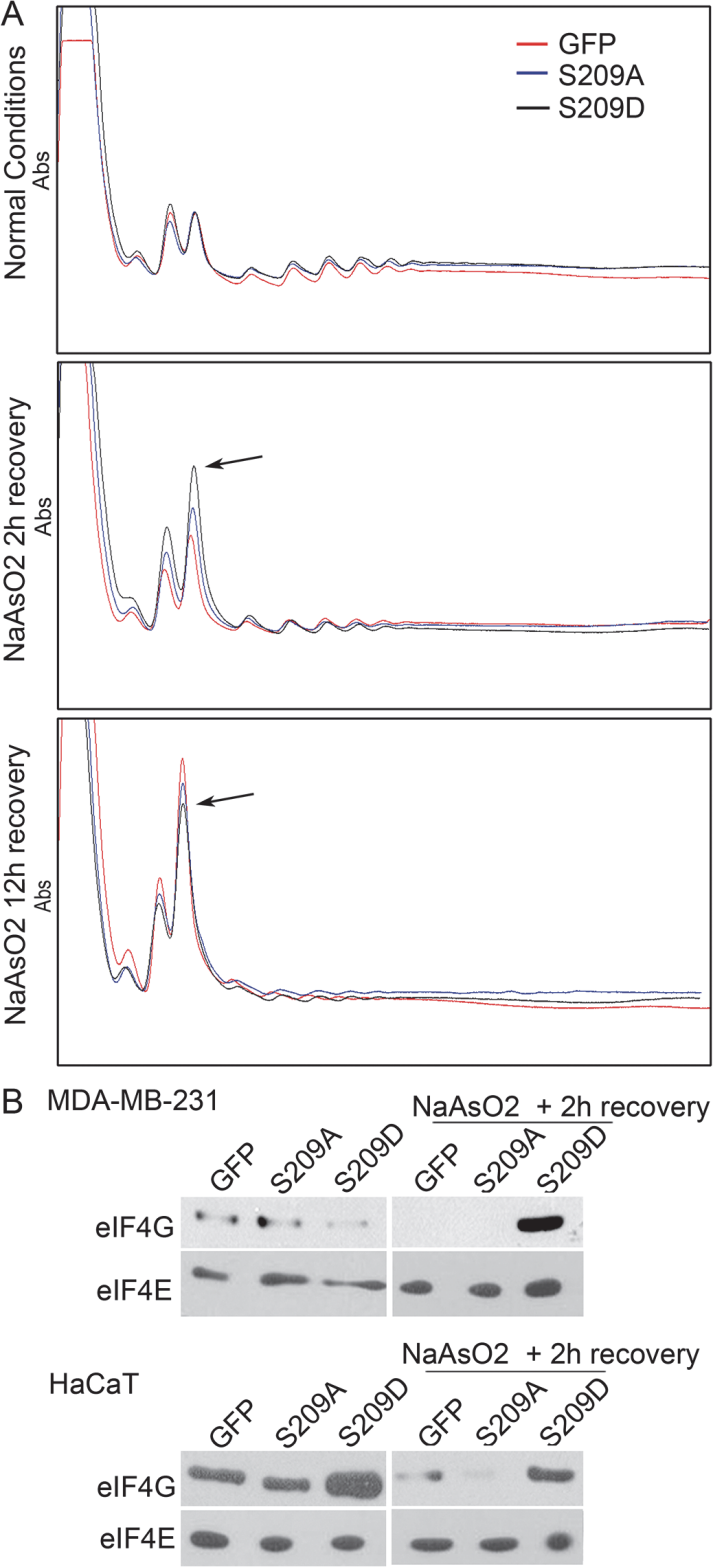
eIF4E-S209D rapidly modulates protein translation after arsenite treatment. Polysome analysis of MDA-MB-231 cells showed that cells expressing eIF4E-S209D, but not-S209A or GFP, induced a peak in the 80S fraction 2 hours after arsenite treatment, indicating translational stalling. Twelve hours after recovery from arsenite treatment, the 80S peak in S209D-expressing cells was more moderate compared with that of S209A- or GFP-expressing cells, indicating a slightly higher level of protein synthesis. B, m7-GTP pull-down assays in normal conditions and 2 hours after arsenite treatment indicate that eIF4E-S209D strongly associates with the mRNA cap in a complex with eIF4G in the recovery period after arsenite treatment, which may allow re-initiation of protein synthesis.

As eIF4E is a rate-limiting regulator of mRNA translation, we sought to analyze changes in mRNA translation and protein synthesis in response to stress. We thus performed polysome profiling to assess overall mRNA translation in MDA-MB-231 cells. Under normal conditions, both phosphomimetic and phospho-dead forms of eIF4E, as well as control GFP-expressing cells, displayed the same polysome profile. Two hours after arsenite treatment, however, the S209D-expressing cells, in comparison to those expressing S209A and GFP, showed an increase in the 80S peak, indicating a rapid impediment to translation. Twelve hours after stress, the 80S peak in S209D-expressing cells was maintained, whereas the S209A- and GFP-expressing cells now displayed an even higher 80S peak (Fig 6A). These results may indicate that cells expressing the phosphomimetic form of eIF4E are able to halt protein synthesis more quickly after a stress situation—but in a moderate manner—presumably to allow selective synthesis of proteins important to survival to continue. m7 GTP pull-down assays, in both MDA-MB-231 and HaCaT cells, clearly showed a more rapid recovery of cap-mRNA binding and association with eIF4G after arsenite stress in cells expressing phosphomimetic eIF4E (Fig 6B). Given that in normal conditions peIF4E binds 4E-T, and that 4E-T and eIF4G compete for the same binding site of eIF4E, we hypothesized that after a stressful situation peIF4E can be released from 4E-T due phosphorylation of 4E-T. Indeed, after arsenite treatment, 4E-T is phosphorylated and this phosphorylation decreases its binding to eIF4E [34]. The release of eIF4E allows its binding with eIF4G and thus the selective synthesis of certain proteins. Concordingly, we observed the release of peIF4E from 4E-T in the cell line MDA-MB-231 two hours after treatment with arsenic (S9A Fig). By immunofluorescence we observed, in both MDA-MB-231 and HaCaT cell lines, that the S209D mutant was released after recovery from stress and no longer associated with 4E-T containing cytoplasmatic bodies (S9B Fig). The significance of these observations clearly merits further investigation.

**Fig 7:**
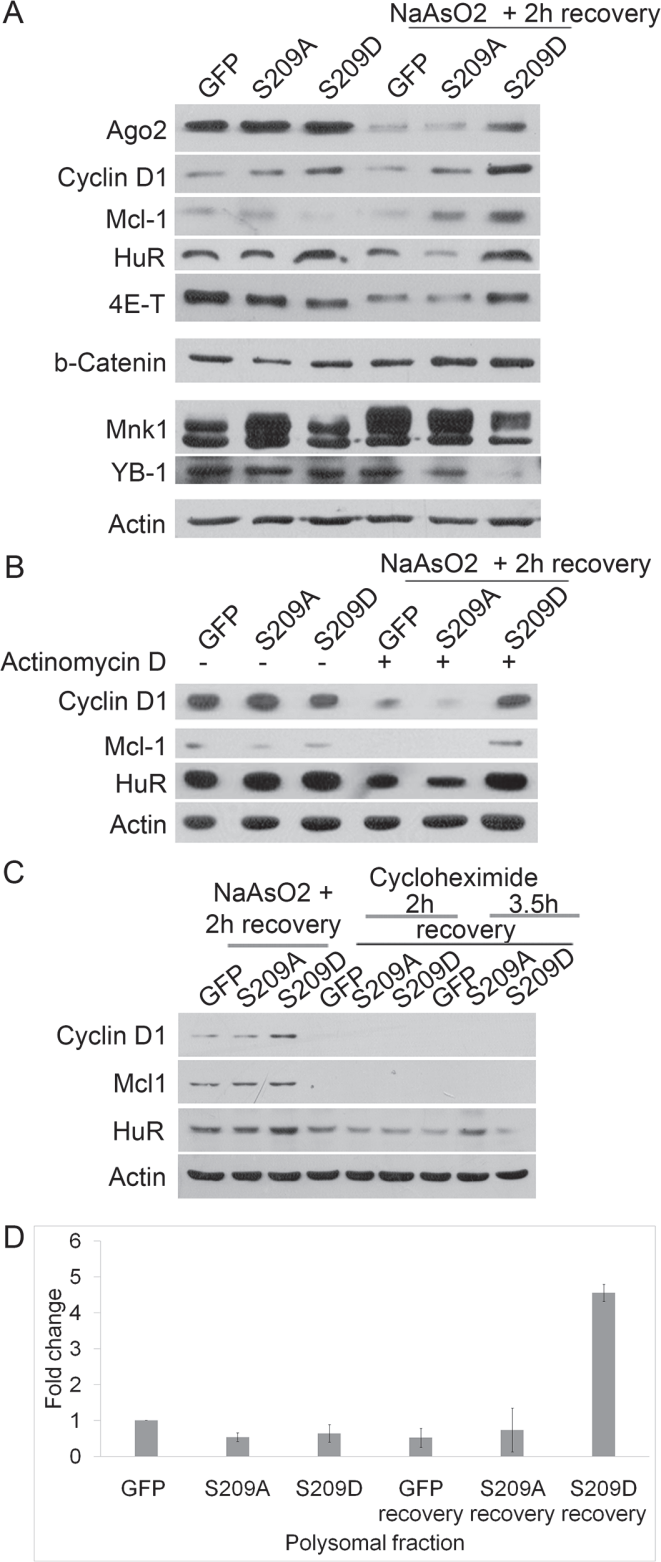
Selective increase in protein synthesis in cells expressing eIF4E-S209D. A, Western blotting analysis showed that eIF4E-S209D was able to maintain the expression levels of some proteins, such as cyclin D, after stress caused by arsenite treatment (compared to S209A- or GFP-expressing cells). Other proteins showed reduced levels or no changes. B, Treatment with actinomycin D for 6 hours in normal conditions and 2 hours after stress indicated that the selective advantage of eIF4E-S209D in maintaining protein expression was due to post-transcriptional events. C, Treatment with cycloheximide after arsenite treatment showed that cyclin D and Mcl-1 are subjected to translational regulation by eIF4E-S209D, rather than modulation of protein stability. D, Quantitative PCR analysis of polysomal RNA indicated that eIF4E-S209D after stress permits a dramatic increase in actively translated cyclin D1 (expressed as a fraction of total mRNA).

To validate whether phosphorylation of eIF4E mediates a selective synthesis of proteins under stress (as shown under normal conditions by other researchers [35]), we screened a number of proteins by western blot under normal conditions and after arsenite treatment. Indeed, S209D expression, but not that of S209A or GFP, caused an increase in the level of some proteins 2 hours after arsenite stress, including proteins associated with resistance to apoptosis (Mcl-1) and cell cycle progression (cyclin D1) whereas other proteins were either maintained or decreased (Fig 7A). To exclude the possibility that S209D works through transcriptional pathways, we treated cells with actinomycin D before and after arsenite stress to block *de novo* transcription. As seen in Fig 7B, S209D prevented the loss of HuR, Mcl-1, and cyclin D1 also in the presence of actinomycin D, strongly suggesting post-transcriptional regulation, possibly by maintenance of mRNA translation. After cycloheximide treatment, blocking protein synthesis, the levels of some proteins, like cyclin D1, Mcl1, and HuR, was significantly reduced after 2 hours of recovery from arsenite treatment (Fig 7C), indicating that phosphomimetic eIF4E indeed acts by maintaining mRNA translation rather than stabilizing these proteins through an alternative mechanism.

To validate this further, we performed Q-PCR analysis of mRNA isolated from polysomal mRNA isolated before or after arsenite treatment. As seen in Fig 7D, cyclin D1 expression clearly increases (relative to total mRNA) after stress specifically in cells overexpressing S209D (Fig 7D). A plausible explanation for these results is that phosphorylation of eIF4E allows for a more rapid re-initiation of protein synthesis following stress.

## Discussion

Recently, control of protein synthesis has emerged as an important concept in the regulation of cancer development, cancer prognosis, and therapeutic response [18, 36–40]. eIF4E, a rate-limiting factor for mRNA translation and a potent oncoprotein, plays a central role in this process. Phosphorylation of the eIF4E residue Ser-209 is mediated by the Mnk1/2 kinases, and, although phosphorylation of eIF4E does not appear to be important for normal development (as shown in various mouse models), it appears to control many of features related to malignancy. The underlying mechanism by which phosphorylation regulates the oncogenic properties of eIF4E is unclear, although several studies have reported increased synthesis of various proteins involved in tumorigenesis, such as Mcl-1 (anti-apoptotic), MMP3 (matrix degradation and invasion), and cyclin D1 (proliferation). The present results shed further light on this process, demonstrating that phosphorylation of eIF4E augments resistance to *in vitro* cellular stress, including oxidative stress, DNA-damaging agents, and nutrient starvation. Indeed, stress appears to be one of the major inducers of eIF4E phosphorylation. This result has profound implications for cancer therapy. Cellular stress is frequently encountered by tumor cells during cancer progression, with sources of stress that include lack of nutrients, cellular crowding, and hypoxia, as well as that encountered during therapeutic interventions, such as during chemo- and radiotherapy. Our results indicate that inhibiting eIF4E phosphorylation is therefore a therapeutic option worth evaluating. Indeed, there are already safe and orally available drugs, such as cercosporamide, that inhibit eIF4E phosphorylation via Mnk1/2 kinases. Our *in vitro* data indicate that blocking eIF4E phosphorylation with CGP 57380 is indeed sufficient to reduce cellular resistance to stress and chemotherapeutic reagents such as cisplatin. Although non-specific effects of CGP 57380 is reported, we believe that our additional experiments using the S209D mutant, Mnk1/2 KO MEFs and Mnk1 shRNA knockdown, all support the idea that CGP 57380 acts via the Mnk1/2-eIF4E pathway fairly specifically and may be useful as a tool to sensitize tumour cells to anti-cancer treatments.

How eIF4E regulates stress resistance remains to be studied in detail, although we have uncovered several possible mechanisms. S209D appears to cause a rapid (within 2 hours) translational block in the 80S complex (polysomal profiles) after stress, followed by a qualitative regulation of synthesis of certain proteins involved in resistance to apoptosis and proliferation, such as cyclin D1, Mcl-1, and MMP3, in line with previous reports [18, 38, 41]. Q-PCR analysis of polysomal mRNA indicate increased translation of these genes. Furthermore, we have demonstrated that resistance to stress requires a direct interaction with the eIF4E-binding protein 4E-T, because knockdown of 4E-T or the use of eIF4E mutants unable to bind 4E-T prevents the stress-protective effect of eIF4E. The role of the phosphorylation of 4E-T and its association with eIF4E/peIF4E has to be investigated further [42]. Importantly, we observed cytoplasmic aggregates of protein in cells expressing the mutant S209D, even at basal conditions. After 90 minutes arsenite treatment and recovery (2 hours), it seems that 4E-T can dissociate from peIF4E, and it is possible, although still speculative, that phosphorylation of 4E-T may be involved in this process. We have also described increased binding of eIF4G to mRNA cap in cells harboring the mutant S209D two hours after arsenite treatment, indicating a release of peIF4E/4E-T and more affinity of peIF4E to the cap. The interplay among 4E-T, eIF4G and 4E-BP1 with eIF4E however, and how these proteins are regulated, needs further investigation.

The association of eIF4E and phospho-eIF4E with stress resistance has been described in several biochemical settings in the literature. For example, Ser-209 of eIF4E is phosphorylated in response to different types of cyto- or genotoxic stresses [43], as well as after treatment with cisplatin [44]. Increased phosphorylation of eIF4E may play a positive role in selectively stimulating synthesis of specific proteins under stress conditions where general protein synthesis is inhibited [43, 45]. In fact, phospho-eIF4E has been associated with enhanced replication of some viruses [46], with playing a significant role in anisomycin-induced translation of CHOP under stress [47], and with exerting a key role in the antiviral host defense [48]. Moreover, in previous work, we showed that high levels of phospho-eIF4E in malignant cells were associated with resistance to an unphosphorylated 4E-BP1 mutant [37]. This mutant binds to eIF4E and exerts a suppressor effect in cell lines [49], but many malignant cell lines with high levels of phospho-eIF4E could escape this inhibition [37].

Finally, the biological role of eIF4E in tumors and its inhibition as a therapeutic target have already been proposed. A phase I clinical trial recently reported mild side-effects, without clinical response, probably due to inefficient *in vivo* knockdown of eIF4E [50]. Other approaches have been developed, including those that use small molecular inhibitors of the binding of eIF4E and eIF4G, such as 4EG-1 [51]. In addition, *in vitro* and *in vivo* studies have shown that knocking down eIF4E enhances sensitivity to anticancer agents, including cisplatin and doxorubicin [41, 44]. Importantly, we show that this effect is mediated by the level of phosphorylated eIF4E and not the total level of eIF4E. Regarding inhibition of the Mnks, data with the inhibitor CGP 57380 have shown a decrease in the cell growth of breast cell lines and BCR-ABL–dependent cell lines [52]. Interestingly, other inhibitors, such as cercosporamide, have been shown to exert antitumor effects *in vivo* and prevent lung metastasis in a melanoma model [53]. In this paper, we show that phospho-eIF4E is clearly associated with resistance to treatment with cisplatin and arsenite and that inhibition of Mnk1/2 significantly increases sensitivity to cisplatin and oxidative stress. Moreover, it is important to realize that treatment with cisplatin and other genotoxic agents increases eIF4E phosphorylation through Mnks and p38. Similarly, treatment with rapamycin enhances eIF4E phosphorylation by activating MNK2a [54].

In summary, because phosphorylation of eIF4E is associated with resistance to cellular stress, with tumor development in mice models, and worse prognosis in human tumors, the use of specific inhibitors of eIF4E phosphorylation may be a promising approach in cancer treatment. We propose combining eIF4E phosphorylation inhibition with other therapeutic approaches, with our data supporting the rationale for combining inhibitors of eIF4E phosphorylation with cisplatin or other DNA-damaging agents. Importantly, lack of eIF4E phosphorylation is not associated with anomalies in mice development and some inhibitors of Mnk1/2, such as cercosporamide, are already employed in several clinical settings to treat diseases, without major clinical side-effects. Moreover, inhibition of this phospho-eIF4E/4E-T complex could be a valuable approach for the development of novel therapeutic strategies.

## Materials and Methods

### Cell culture

Immortalized keratinocytes, HeLa cells (CCL-2, ATCC (American Type Culture Collection), Middlesex, UK), the breast carcinoma cell lines MDA-MB-231 (CRM-HTB-26, ATCC) and MDA-MB-468 (HTB-132, ATCC) [37], and mouse embryonic fibroblasts (MEFs) and MEFs derived from Mnk1/2 KO [55] mice were grown in standard DMEM (Dulbecco's modified Eagle’s medium) growth medium supplemented with 10% fetal calf serum and 1% penicillin/streptomycin. Approved by the *Vall d’Hebron Institut de Recerca* ethical committee (PR(SC)92/2011). MDA-MB-231 cells were treated overnight with 40 μM of MNK inhibitor (CGP 57380; Tocris Bioscience).

### Cellular stress

Either 2,000 (MEF WT and MEF KO) or 5,000 cells/well (HaCaT and MDA-MB-231) were seeded in 96-well plates and exposed to different types of stress: 250 μM NaAsO_2_ for 90 minutes, 33 μM cisplatin (CDDP) for 3 hours, or nutrient starvation (glucose and glutamine deprivation) for 24 hours. After stress was induced, the medium was changed to normal medium.

### Statistical analysis

Student’s t-test has been done for all the statistical analysis in triplicate experiments, accepting statistical significance a P-value less than 0.05. P-value is shown in each graph.

### Plasmid constructs

pLPCX-S209A-eIF4E, pLPCX-S209D-eIF4E, pLPCx-W73A, pLPCX-W73AS209D, and pLPKo-sh4E-T constructs were kindly provided by Dr. N. Sonenberg (McGill University, Montreal, Canada). Both eIF4E mutants contain one Myc tag. Human pMV7.2-sheIF4E3UTR construct were kindly provided by Dr. Sonia V Del Rincón (Segal Cancer Centre, Montreal, Canada).

### Cell transfection and retroviral transduction

Retroviral production and infection were essentially performed as previously described [56] except that Phoenix packaging cells were transfected with JetPEI according to the manufacturer’s protocol. Viral production and infection were performed at 37°C. Twenty-four hours after the second infection, cells were selected with puromycin (0.7 μg/mL for MDA-MB-231 and 1.5 μg/mL for HaCaT cells) for 3 days to eliminate uninfected cells.

### Anchorage-dependent clonogenic assay

Ten thousand cells were plated in 100-mm plates and cultured for 12 days prior to fixation with 4% paraformaldehyde (Sigma-Aldrich, Taufkirchen, Germany). After fixation, cells were washed with phosphate-buffered saline (PBS) and stained with 0.1% crystal violet (Sigma) for 30 minutes, rinsed thoroughly, and dried. Each cell type was plated in duplicate for each experiment.

### MTT assay

MTT (3-[4,5-dimethylthiazol-25-yl]-2.5-diphenyltetrazolium bromide; Sigma) was added to the medium to a final concentration of 0.5 mg/mL and incubated for 4 hours at 37°C. The medium was then removed and 0.2 mL DMSO was added. Absorbance was measured at 590 nm by using a Synergy spectrophotometer (Biotek). Readings were taken 0, 24, 48, 72, and 96 hours after cell treatment.

### Caspase assay

To measure caspase-3 and -7 activity, the Caspase-Glo 3/7 Assay (Promega) was used. Five thousand cells in 200 uL were seeded in white-walled 96-well plates and treated with 250 μM NaAsO_2_ for 90 minutes. Then 100 μL Caspase-Glo 3/7 reagent was added to each well and the cells were incubated at room temperature for 1 hour. Luminescence was measured in the Synergy Mx Monochromator-Based Multi-Mode Microplate Reader.

### Immunofluorescence

Cells were plated in 24-well plates, fixed for 30 minutes with 4% formaldehyde in PBS, blocked with 5% bovine serum albumin (BSA)/PBS, and permeabilized with 0.5% Triton X-100 in PBS for 1 hour. Cells were incubated with primary antibodies for 2 hours at room temperature and washed three times with PBS both before and after incubation with Alexa Fluor 488- or 594-conjugated secondary antibodies (1:200; Molecular Probes) for 1 hour at room temperature. Cells were mounted in 50% glycerol in PBS. Images were taken with an Espectral FV1000 Olympus confocal microscope.

### Western blot analysis

Western blotting was performed as previously described [37]. The primary antibodies used were as follows: anti-Myc-tag antibody (#2276; 1:1000), anti-peIF4E Ser-209 (#9741; 1:1000), anti-Mnk1 C4C1 (#2195; 1:1000), anti-eIF4G (#2498; 1:1000), anti-YB1 (#4202; 1:1000), anti-mTOR 7C10 (#2983; 1:1000), anti-β-catenin (#9562; 1:1000; all from Cell Signaling); anti-EIF4ENIF1 (three different antibodies: 1:500; Sigma; 1:1000 Cell Signaling; 1:1000 Abnova); anti-Cyclin D1 M-20 (1:1000; Santa Cruz Biotechnology); anti-MCL1 Y37 (1:500), anti-Ago2 (1:500), anti-GAPDH 6C5 (1:2000; all from Abcam); and anti-HuR (1:500; Millipore). Anti-actin CP01 (1:500; Calbiochem, Darmstadt, Germany) was used as a loading control. The secondary antibodies used were donkey anti-rabbit IgG-HRP (NA9340; 1:2000) and donkey anti-mouse IgG-HRP (NA9340; 1:2000; both from Amersham Pharma-Biotech, Uppsala, Sweden). Bound antibodies were visualized with an enhanced chemiluminescence detection kit (Amersham Pharma-Biotech).

### Immunoprecipitation

Cells were harvested in lysis buffer (250 mM Tris-HCl, 150 mM NaCl, 1% Triton X-100, and 5 mM EDTA). Lysed cells were incubated with anti-Myc tag antibody (diluted 1:2000), eIF4E antibody (diluted 1:500), or 4E-T antibody (diluted 1:500) overnight at 4°C. The following day Protein G Sepharose 4 Fast Flow (17-0618-01; Amersham Pharma-Biotech) was added and the solution was gently mixed for 1 hour at 4°C. Samples were centrifuged at 3000 rpm for 3 minutes. The pellet was washed 3 times with 1 mL lysis buffer and resuspended in 50 μL sample buffer (1% SDS, 100 mM DTT, 50 mM Tris, pH7.5). The suspension was heated to 95°C for 5 minutes and centrifuged at 3000 rpm for 3 minutes to remove the beads.

### 7-methyl-GTP-Sepharose pull-down assays

Cells were lysed in lysis buffer (50 mM HEPES, pH 7.5, 150 mM NaCl, 1% Triton X-100, 1 mM EDTA, 10% glycerol). Cell extracts were incubated overnight with anti-Myc tag primary antibody (diluted 1:2000). 7-methyl-GTP-Sepharose or control Sepharose (GE Healthcare) was added for 1 hour at 4°C under constant shaking. Beads were washed three times with lysis buffer and absorbed proteins were eluted in SDS-PAGE sample buffer.

### Polysome profiling

For polysome analysis, 400 mg of cell lysate was frozen in liquid nitrogen and then resuspended in 1 mL of polysome extraction lysis buffer (1.5 mM KCl, 2.5 mM MgCl_2_, 5.0 mM Tris, pH 7.4, 1.0% Triton X-100, 1.0% Na-deoxycholate, a protease inhibitor mix, 100 U/mL RNase OUT, and 100 μg/mL cycloheximide). Cell extracts were analyzed in a 10%–50% sucrose gradient (200 mM HEPES, 1 M KCl, 50 mM MgCl_2_, 100 μg/mL cycloheximide, a protease inhibitor mix, and 200 U/mL RNase OUT). Extracts were centrifuged in an SW41Ti rotor for 2 hours and 30 minutes at 37000 rpm at 4°C. Gradients were sampled by using an ISCO UV detector.

## Supporting Information

S1 Fig Clonogenic cell survival assay. A, Graphs representing the number of colonies in MDA-MB-231 and HaCaT cells from the clonogenic assay under normal conditions and after arsenite pre-treatment. The expression of eIF4E-S209D increased the number of colonies in both conditions. B, HaCaT and MDA-MB-231 cells expressing eIF4E-S209D and eIF4E-WT showed similar ability to form colonies under normal conditions. C, Graphs representing the number of colonies. The number of colonies was higher in the phosphomimetic mutant than in the phospho-dead eIF4E, and similar to the wild type. D, MDA-MB-468 cells expressing eIF4E-S209D showed greater clonogenic colony formation ability under normal conditions than-S209A– or GFP–expressing cells.
(TIF)

S2 Fig MTT cell recovery assay. MTT assays in MDA-MB-468 and HeLa cell lines after arsenite treatment and nutrient starvation indicated significantly faster recovery after stress in cells expressing eIF4E-S209D than in those expressing-S209A or GFP. * = P<0.05 and ** = P<0.01 compared to control, n = 3.
(TIF)

S3 Fig Growth and survival after knocking down endogenous eIF4E. A, MDA-MB-231 stably expressing either GFP or S209A or S209D mutants of eIF4E were transfected with a short hairpin plasmid targeting the endogenous 3’UTR of eIF4E. Western blot clearly shows knock-down of endogenous eIF4E without affecting exogenous eIF4E. B, MTT assays after arsenite treatment suggest reduced endogenous eIF4E significantly reduces the recovery capacity after arsenite treatment. * = P<0.05, ** = P<0.01 and *** = P<0.001 compared to control, n = 3. C, overexpression of S209D completely rescues cells and allow recovery from arsenite and CDDP. S209A also moderately improves recovery in the context of reduced endogenous eIF4E and arsenite treatment, perhaps as it may substitute for some functions of endogenous (unphosphorylated eIF4E).
(TIF)

S4 Fig Growth and survival after Mnk1/2-inhibition. A, MTT assay in MEF Mnk1/2 KO with and without CGP57380 treatment and with arsenite treatment indicates that the effect of CGP 57380 is mainly due to inhibition of the Mnk1/2 pathway. B, Mnk1 depletion in normal conditions in MDA-MB-231 using a lentiviral shMnk1 construct. C, MTT assay in MDA-MB-231 pre-treated with arsenite. Both shMnk1 knockdown and CGP 57380 treatment displayed a similar reduction in recovery after arsenite treatment, inhibiting the recovery.
(TIF)

S5 Fig Immunofluorescence analysis of eIF4E and 4E-T in cytoplasmic bodies. A, Higher magnification of immunofluorescence analysis of MDA-MB-231 expressing S209D (20X and 40X). 4E-T antibody Sigma HPA001619 B, Immunofluorescence analysis of HaCaT cells expressing either S209A or S209D mutants of eIF4E under normal conditions indicated the specific spontaneous formation of cytoplasmic bodies in S209D-expressing cells. These bodies partially colocalized with 4E-T. 4E-T antibody Cell Signaling 2297, unspecific staining is observed in the nucleus. C, Immunofluorescence analysis of MDA-MB-231 after arsenite treatment to increase the levels of endogenous peIF4E, colocalization of peIF4E with 4E-T in cytoplasmic bodies. 4E-T antibody Abnova H00056478.
(TIF)

S6 Fig Immunoprecipitation assay with eIF4E antibody in MEF WT and MEF Mnk1/2 KO indicates more affinity of peIF4E to 4E-T in normal conditions. (TIF)

S7 Fig 4E-T depletion in normal conditions in eIF4E mutants. MDA-MB-231 stably expressing either GFP or S209A or S209D mutants of eIF4E were cotransfected with sh4E-T. Endogenous 4E-T levels were reduced in all three cases.
(TIF)

S8 Fig The eIF4E mutation W73A disrupts the interaction with 4E-T. Immunoprecipitation assays with anti-Myc-tag antibodies against eIF4E-W73A and—W73A/S209D confirmed previous findings that the W73A mutation prevents the direct interaction with 4E-T.
(TIF)

S9 Fig Interaction between endogenous p-eIF4E and 4E-T. A, immunoprecipitation of endogenous peIF4E in MDA-MB-231. Two hours after arsenite treatment there are no binding of 4E-T to peIF4E. B, two hours after arsenite treatment in MDA-MB-231 and HaCaT cell lines, eIF4E-S209D mutant realize the binding with 4E-T.
(TIF)
