# Supplementary figures and images for: Phosphorylation of eIF4E Confers Resistance to Cellular Stress and DNA-Damaging Agents through an Interaction with 4E-T: A Rationale for Novel Therapeutic Approaches
Source: PLoS One. 2015 Apr 29;10(4):e0123352. doi: 10.1371/journal.pone.0123352 (PMC4414544; doi:10.1371/journal.pone.0123352)

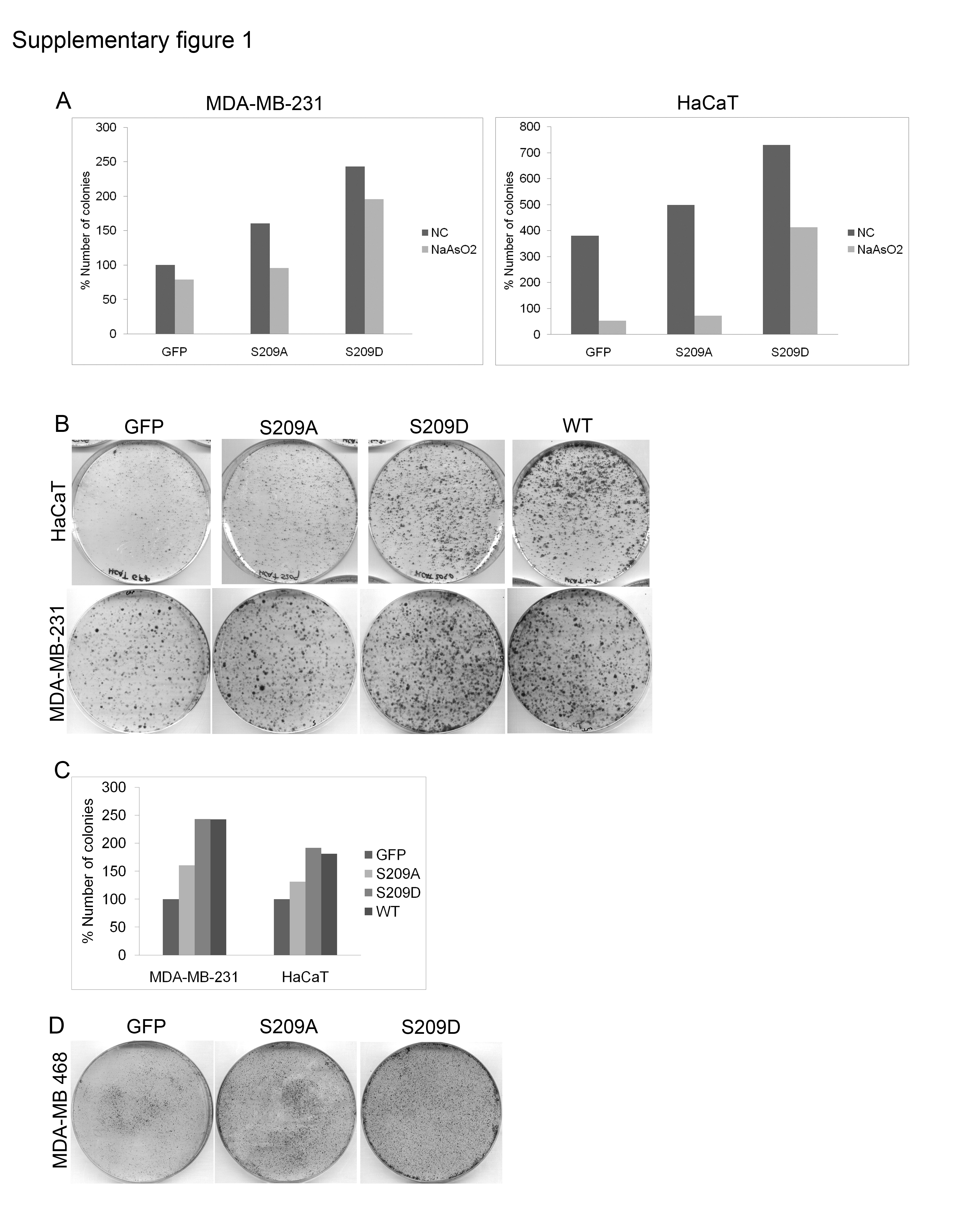

Supplement: S1 Fig — A, Graphs representing the number of colonies in MDA-MB-231 and HaCaT cells from the clonogenic assay under normal conditions and after arsenite pre-treatment. The expression of eIF4E-S209D increased the number of colonies in both conditions. B, HaCaT and MDA-MB-231 cells expressing eIF4E-S209D and eIF4E-WT showed similar ability to form colonies under normal conditions. C, Graphs representing the number of colonies. The number of colonies was higher in the phosphomimetic mutant than in the phospho-dead eIF4E, and similar to the wild type. D, MDA-MB-468 cells expressing eIF4E-S209D showed greater clonogenic colony formation ability under normal conditions than-S209A– or GFP–expressing cells. (TIF) [file pone.0123352.s001.tif]

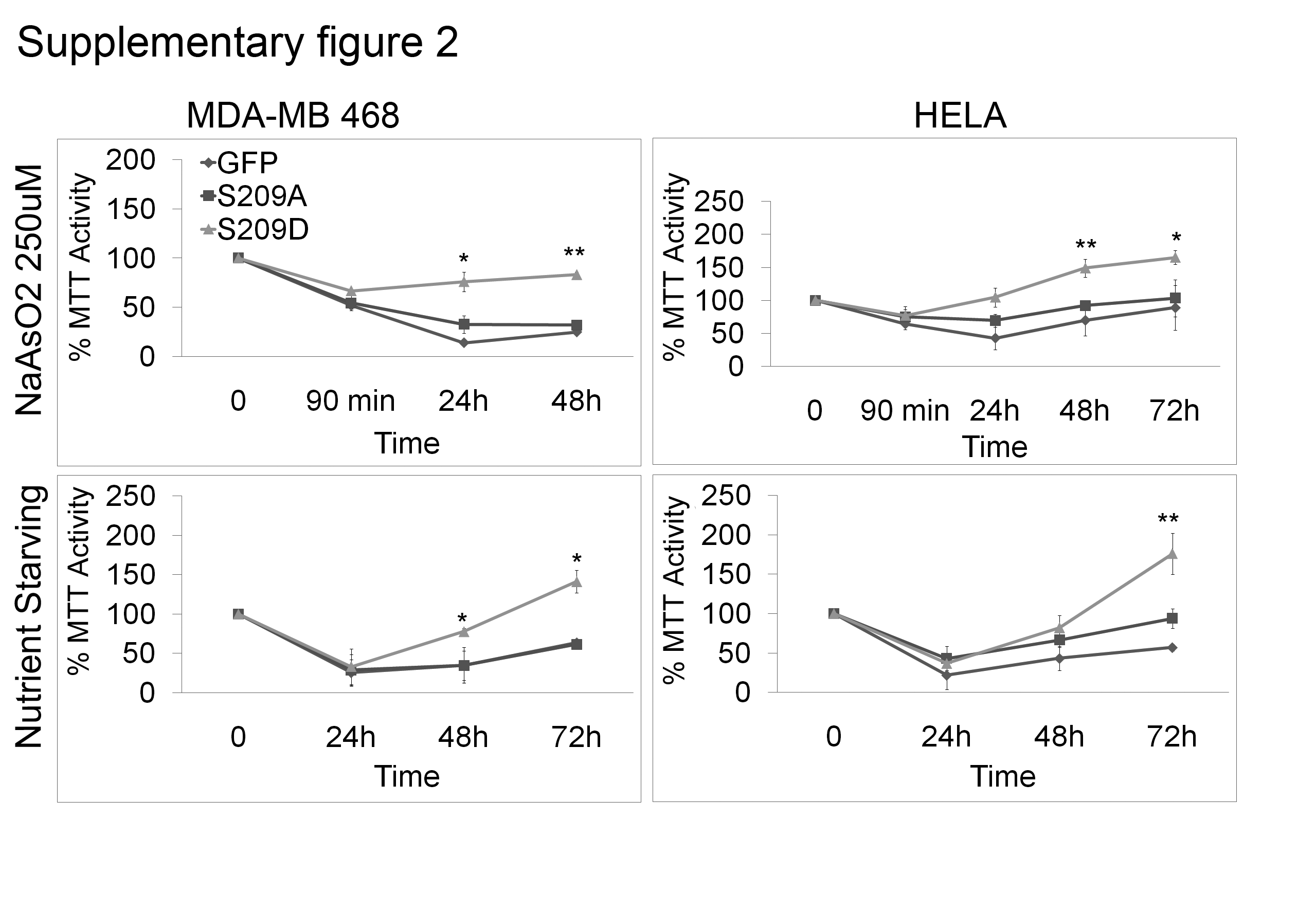

Supplement: S2 Fig — MTT assays in MDA-MB-468 and HeLa cell lines after arsenite treatment and nutrient starvation indicated significantly faster recovery after stress in cells expressing eIF4E-S209D than in those expressing-S209A or GFP. * = P<0.05 and ** = P<0.01 compared to control, n = 3. (TIF) [file pone.0123352.s002.tif]

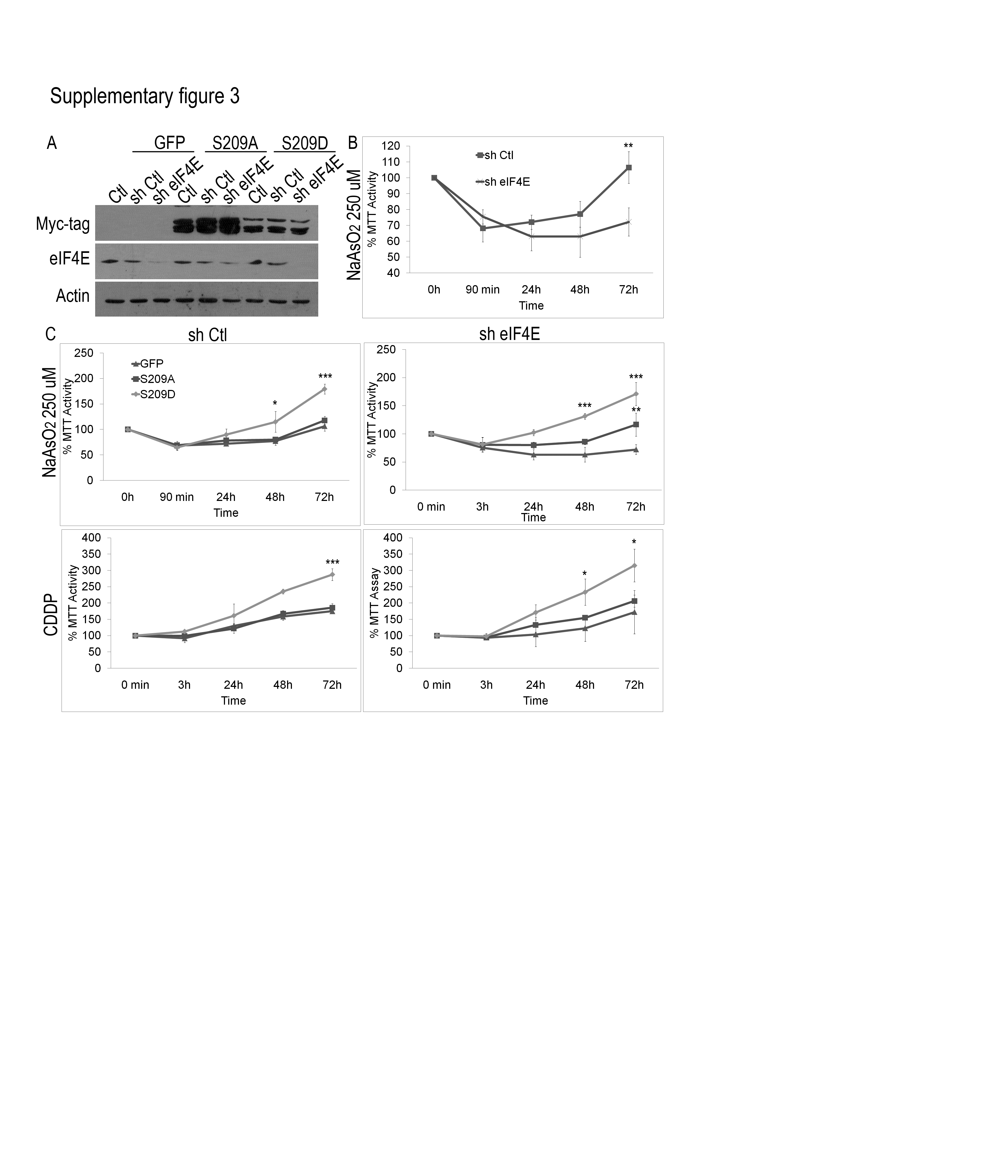

Supplement: S3 Fig — A, MDA-MB-231 stably expressing either GFP or S209A or S209D mutants of eIF4E were transfected with a short hairpin plasmid targeting the endogenous 3’UTR of eIF4E. Western blot clearly shows knock-down of endogenous eIF4E without affecting exogenous eIF4E. B, MTT assays after arsenite treatment suggest reduced endogenous eIF4E significantly reduces the recovery capacity after arsenite treatment. * = P<0.05, ** = P<0.01 and *** = P<0.001 compared to control, n = 3. C, overexpression of S209D completely rescues cells and allow recovery from arsenite and CDDP. S209A also moderately improves recovery in the context of reduced endogenous eIF4E and arsenite treatment, perhaps as it may substitute for some functions of endogenous (unphosphorylated eIF4E). (TIF) [file pone.0123352.s003.tif]

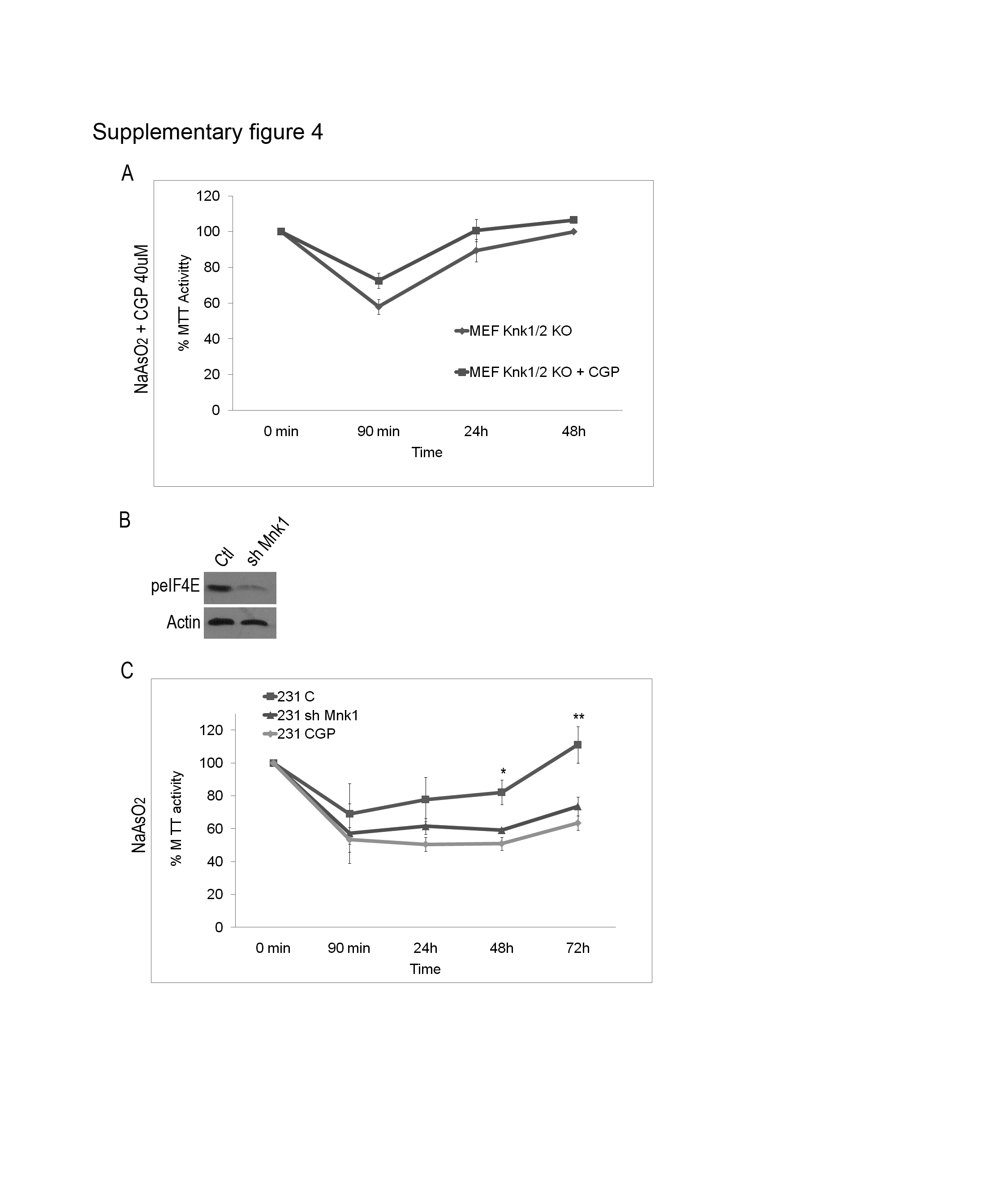

Supplement: S4 Fig — A, MTT assay in MEF Mnk1/2 KO with and without CGP57380 treatment and with arsenite treatment indicates that the effect of CGP 57380 is mainly due to inhibition of the Mnk1/2 pathway. B, Mnk1 depletion in normal conditions in MDA-MB-231 using a lentiviral shMnk1 construct. C, MTT assay in MDA-MB-231 pre-treated with arsenite. Both shMnk1 knockdown and CGP 57380 treatment displayed a similar reduction in recovery after arsenite treatment, inhibiting the recovery. (TIF) [file pone.0123352.s004.tif]

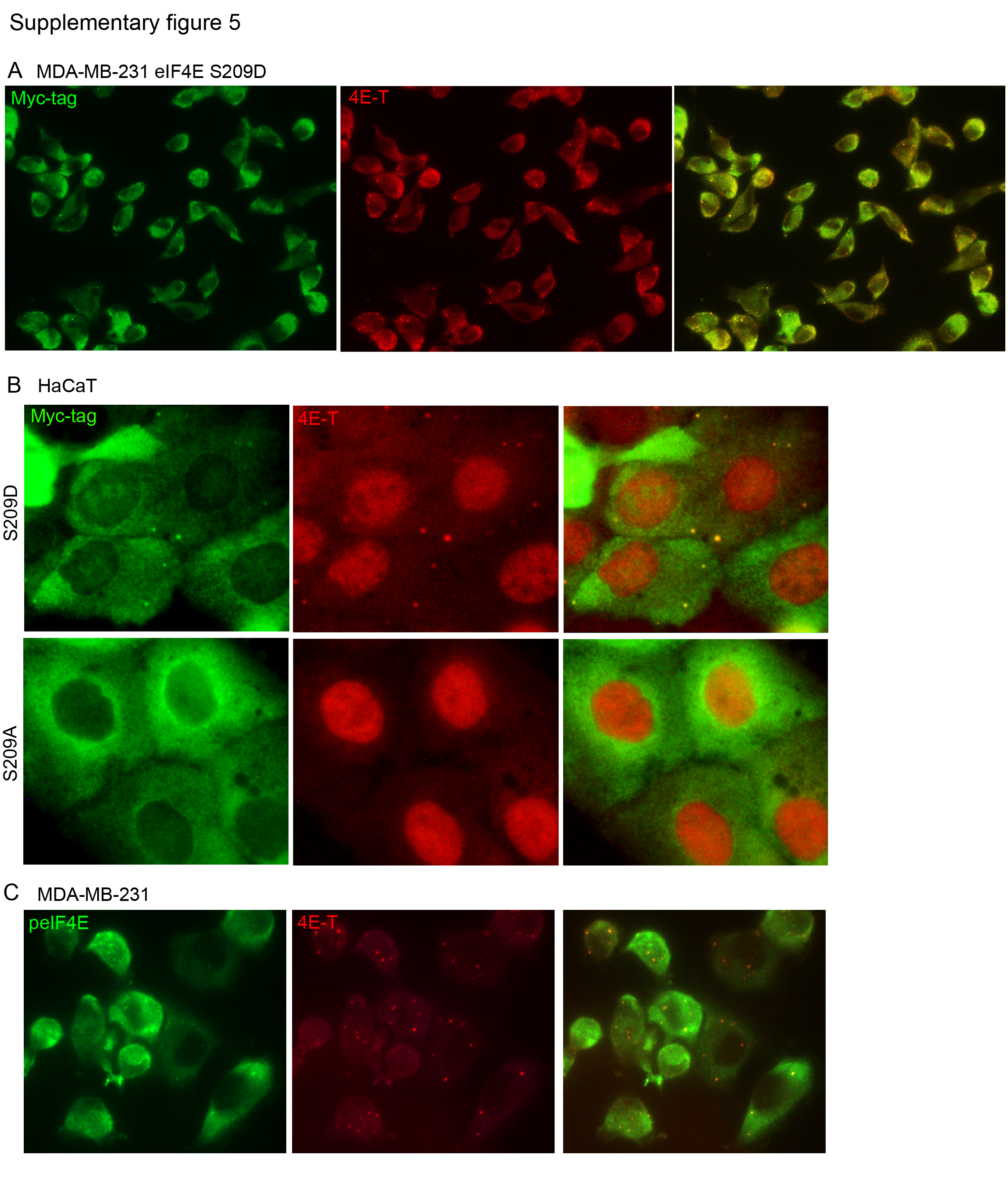

Supplement: S5 Fig — A, Higher magnification of immunofluorescence analysis of MDA-MB-231 expressing S209D (20X and 40X). 4E-T antibody Sigma HPA001619 B, Immunofluorescence analysis of HaCaT cells expressing either S209A or S209D mutants of eIF4E under normal conditions indicated the specific spontaneous formation of cytoplasmic bodies in S209D-expressing cells. These bodies partially colocalized with 4E-T. 4E-T antibody Cell Signaling 2297, unspecific staining is observed in the nucleus. C, Immunofluorescence analysis of MDA-MB-231 after arsenite treatment to increase the levels of endogenous peIF4E, colocalization of peIF4E with 4E-T in cytoplasmic bodies. 4E-T antibody Abnova H00056478. (TIF) [file pone.0123352.s005.tif]

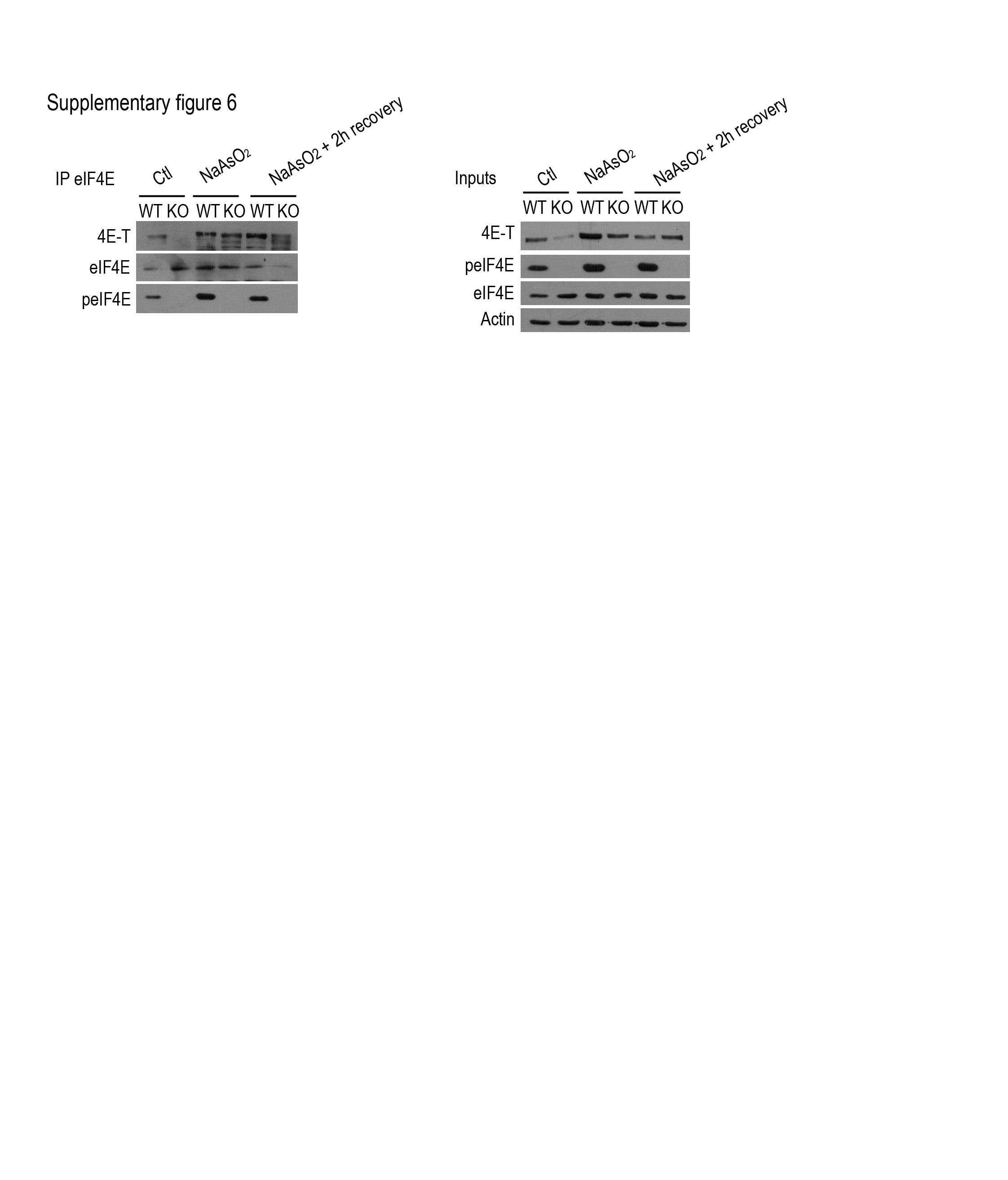

Supplement: S6 Fig — (TIF) [file pone.0123352.s006.tif]

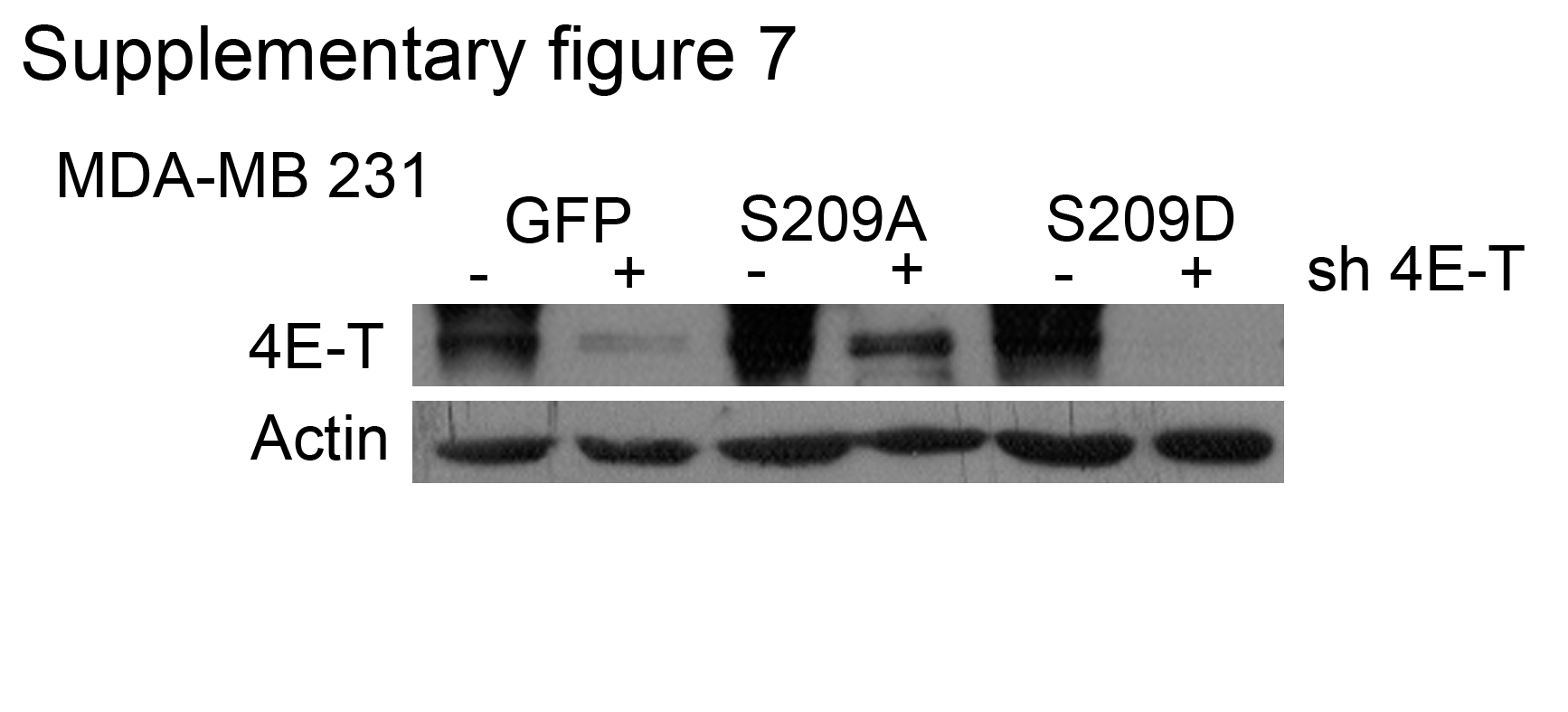

Supplement: S7 Fig — MDA-MB-231 stably expressing either GFP or S209A or S209D mutants of eIF4E were cotransfected with sh4E-T. Endogenous 4E-T levels were reduced in all three cases. (TIF) [file pone.0123352.s007.tif]

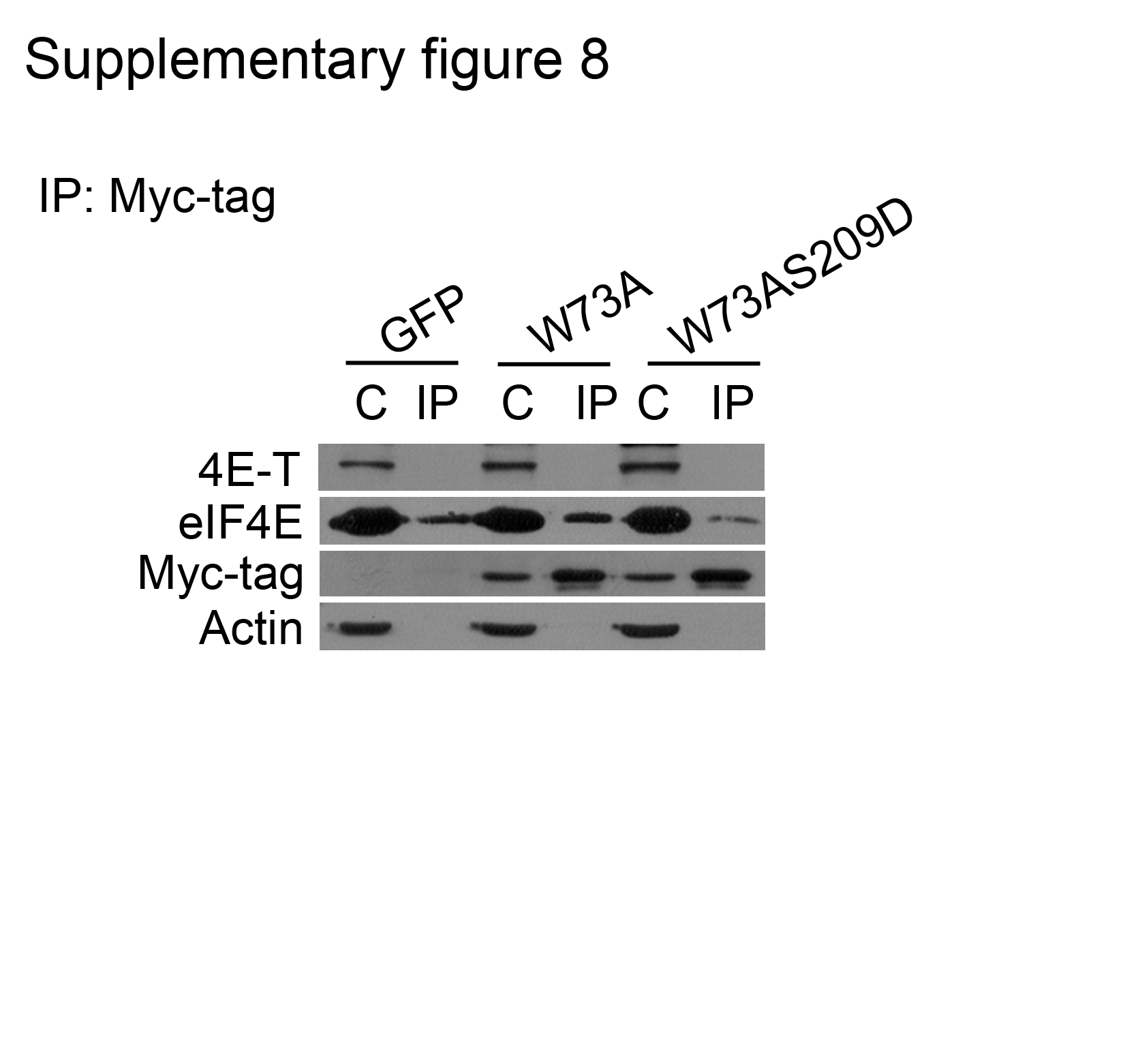

Supplement: S8 Fig — Immunoprecipitation assays with anti-Myc-tag antibodies against eIF4E-W73A and—W73A/S209D confirmed previous findings that the W73A mutation prevents the direct interaction with 4E-T. (TIF) [file pone.0123352.s008.tif]

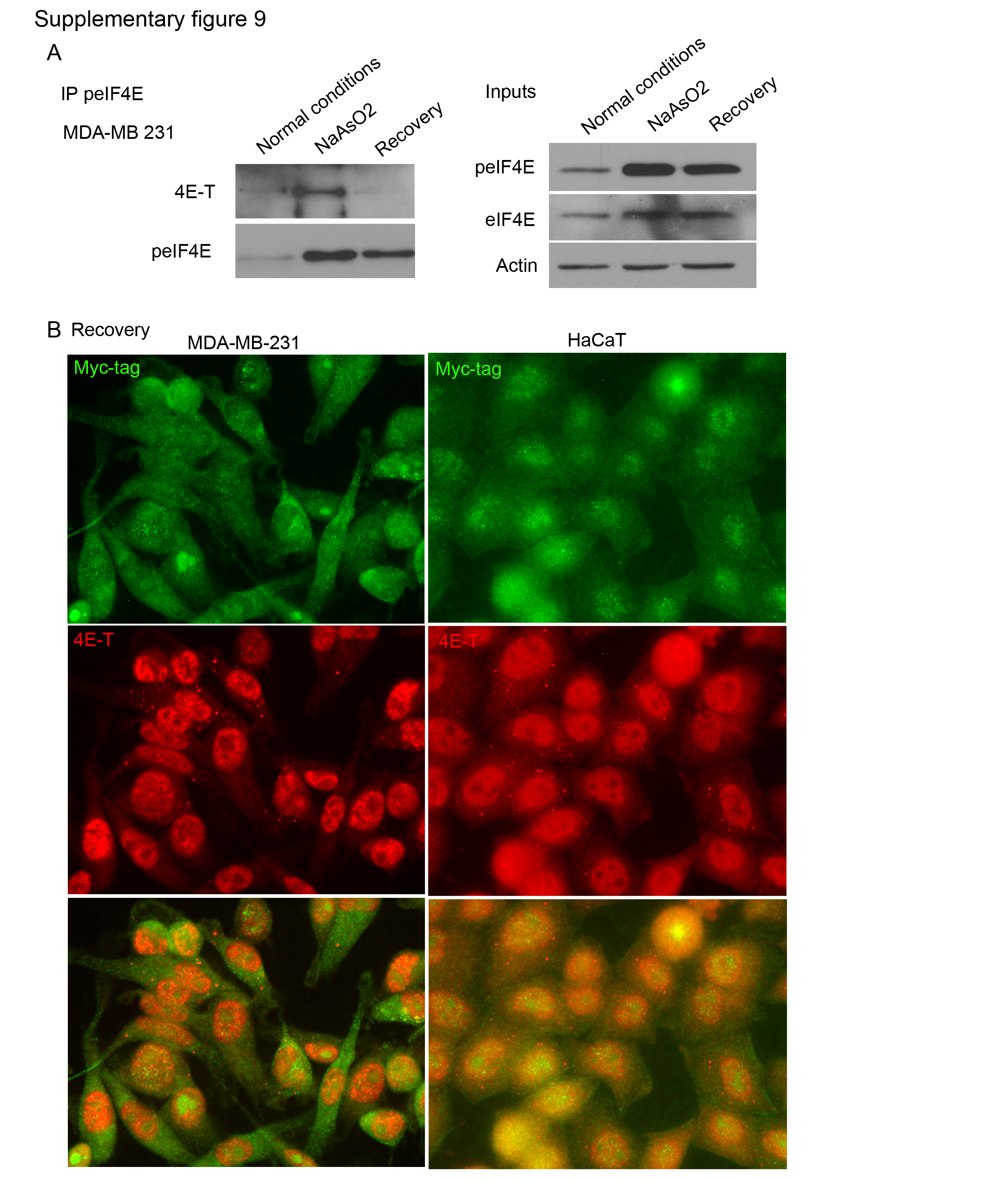

Supplement: S9 Fig — A, immunoprecipitation of endogenous peIF4E in MDA-MB-231. Two hours after arsenite treatment there are no binding of 4E-T to peIF4E. B, two hours after arsenite treatment in MDA-MB-231 and HaCaT cell lines, eIF4E-S209D mutant realize the binding with 4E-T. (TIF) [file pone.0123352.s009.tif]
